# No Evidence for Automatic Remapping of Stimulus Features or Location Found with fMRI

**DOI:** 10.3389/fnsys.2016.00053

**Published:** 2016-06-13

**Authors:** Mark D. Lescroart, Nancy Kanwisher, Julie D. Golomb

**Affiliations:** ^1^Helen Wills Neuroscience Institute, University of CaliforniaBerkeley, CA, USA; ^2^McGovern Center for Brain Research, Massachusetts Institute of TechnologyCambridge, MA, USA; ^3^Department of Psychology, Center for Cognitive and Brain Sciences, Ohio State UniversityColumbus, OH, USA

**Keywords:** remapping, feature remapping, fMRI, visual cortex, spatial updating

## Abstract

The input to our visual system shifts every time we move our eyes. To maintain a stable percept of the world, visual representations must be updated with each saccade. Near the time of a saccade, neurons in several visual areas become sensitive to the regions of visual space that their receptive fields occupy after the saccade. This process, known as remapping, transfers information from one set of neurons to another, and may provide a mechanism for visual stability. However, it is not clear whether remapping transfers information about stimulus features in addition to information about stimulus location. To investigate this issue, we recorded blood-oxygen-level dependent (BOLD) functional magnetic resonance imaging (fMRI) responses while human subjects viewed images of faces and houses (two visual categories with many feature differences). Immediately after some image presentations, subjects made a saccade that moved the previously stimulated location to the opposite side of the visual field. We then used a combination of univariate analyses and multivariate pattern analyses to test whether information about stimulus location and stimulus features were remapped to the ipsilateral hemisphere after the saccades. We found no reliable indication of stimulus feature remapping in any region. However, we also found no reliable indication of stimulus location remapping, despite the fact that our paradigm was highly similar to previous fMRI studies of remapping. The absence of location remapping in our study precludes strong conclusions regarding feature remapping. However, these results also suggest that measurement of location remapping with fMRI depends strongly on the details of the experimental paradigm used. We highlight differences in our approach from the original fMRI studies of remapping, discuss potential reasons for the failure to generalize prior location remapping results, and suggest directions for future research.

## Introduction

Eye movements present a fundamental challenge for visual perception. The input to our visual system shifts abruptly every time we move our eyes, up to three times per second in natural vision. In order to support perception of a stable world, the visual system needs some way to reconcile input that arrives before and after each eye movement.

An influential theory suggests that the brain aligns the visual world and its neural representation by “remapping” neural activity around the time of saccades. In a seminal study, Duhamel et al. (1992) found that the receptive fields of many neurons in macaque lateral intraparietal area (LIP) become responsive to stimuli at the locations their receptive fields will occupy after a saccade. The authors suggested that this process constitutes a “remapping of the stimulus from the coordinates of the initial fixation to those of the intended fixation” (Duhamel et al., 1992).

Remapping has also been demonstrated in the frontal eye fields (FEF; Umeno and Goldberg, 1997), early visual areas including V1, V2, V3, and V3A (Nakamura and Colby, 2002), and the superior colliculus (Walker et al., 1995). Thus in a network of areas throughout the brain, remapped activity seems to provide an automatic prediction of the sensory consequences of eye movements (but see also Zirnsak et al., 2011, 2014; Neupane et al., 2016).

However, it is not clear whether remapped activity encodes feature information in addition to location information (Melcher and Colby, 2008; Cavanagh et al., 2010; Zirnsak and Moore, 2014). The question of what remaps is critical for understanding the function of remapping. Does remapping transfer feature information? Or does remapping transfer only “attentional pointers” to salient or otherwise behaviorally relevant locations in the visual field that have been prioritized for further processing (Cavanagh et al., 2010; Rolfs and Szinte, 2016)? Remapping of stimulus location and features would make remapping a plausible mechanism for comparing visual input across saccades, and thus strengthen the argument that remapping is involved in perceptual stability. If feature information does not remap, remapping is less likely to be critical for perceptual stability, but may still be useful for localization and tracking of objects. It is also important to determine whether remapping happens automatically or whether it occurs only under specific circumstances.

Several recent studies in psychophysics and neurophysiology have searched for feature remapping with mixed results. Melcher (2007) demonstrated that behavioral tilt aftereffects can occur across different retinal locations following an eye movement, suggesting that orientation information can be remapped. However, follow-up work has suggested that the effects Melcher observed were due to the spread of attention, not remapping of feature information (Knapen et al., 2009, 2010), and that tilt aftereffects are stronger near the saccade target than in the remapped location (Zirnsak et al., 2011). Other studies have pointed to transsaccadic feature integration as evidence for feature remapping, although these findings have also produced mixed results and interpretations (Irwin et al., 1988; Hayhoe et al., 1991; Melcher and Morrone, 2003; Prime et al., 2006; Demeyer et al., 2011; Harrison and Bex, 2014; Oostwoud Wijdenes et al., 2015).

In neurophysiology, most studies have focused on location remapping, although a few recent studies have provided tentative evidence for feature remapping. Subramanian and Colby (2014) found that remapped responses in a small fraction of neurons in macaque LIP show shape selectivity. However, in most cases the remapped shape selectivity did not match the shape selectivity observed with direct stimulation of the same neurons. O’Herron and von der Heydt (2013) recently found remapping of border ownership in V2 cells. However, border ownership tuning may depend on feedback from higher-order areas (Zhou et al., 2000), and border ownership is associated with mechanisms for the spread of attention (Qiu et al., 2007). Thus it is still unclear whether remapping automatically transfers feature information or merely attentional pointers (Cavanagh et al., 2010).

In the current study, we use functional magnetic resonance imaging (fMRI) to search for automatic stimulus feature remapping. We use fMRI because it allows simultaneous measurement of many brain regions, and fMRI pattern classification analyses can exploit the data available in each region to distinguish different classes of stimuli. fMRI cannot distinguish functional signals that precede and follow eye movements by a few hundred milliseconds. However, many neurons show a form of remapping dubbed “memory trace remapping, ” in which they respond after a saccade to a stimulus that was only present before the saccade. This activity can be understood as a slightly delayed prediction of the sensory consequences of an eye movement, and has been demonstrated using single unit electrophysiology (Duhamel et al., 1992; Nakamura and Colby, 2002; Heiser and Colby, 2006), EEG (Bellebaum et al., 2005; Bellebaum and Daum, 2006), and fMRI (Merriam et al., 2003, 2007). Indeed, more neurons exhibit memory trace remapping than anticipatory remapping (Duhamel et al., 1992; Nakamura and Colby, 2002; Heiser and Colby, 2006).

In the fMRI memory trace remapping paradigm designed by Merriam et al. (2003, 2007), subjects saw a circle of light that flashed on and off several times in 1 s in a peripheral location. In some trials, subjects were cued to make an eye movement immediately after the stimulus was extinguished. In the absence of an eye movement, the stimulus only activated regions in the contralateral hemisphere. However, after eye movements that would have brought the stimulus memory trace into the opposite visual hemifield, fMRI responses were observed in the other hemisphere (which was never directly stimulated). This demonstration was taken as evidence that information about the stimulus location was remapped across hemispheres. However, the studies by Merriam et al. could not determine whether only the location of the stimulus was remapped, or whether stimulus feature information might also be remapped. In the current study, we modified the paradigm of Merriam et al. (2007) by presenting images of faces and houses before the saccade, and using multivariate pattern analysis (MVPA) to test whether there was information about stimulus features in the remapped response.

We conducted three fMRI experiments using variants of this paradigm, and we examined multiple candidate brain regions (visual areas V1–V4, Lateral Occipital cortex [LO], Occipital Place Area [OPA], Parahippocampal Place Area [PPA], Occipital Face Area [OFA], and Fusiform Face Area [FFA]). We found no evidence for automatic feature remapping in any of these regions. However, we also unexpectedly failed to find evidence for remapping of stimulus location, even though our experimental paradigm was similar to the original fMRI remapping paradigm (Merriam et al., 2003, 2007). Thus, our results cannot support a strong conclusion about whether or not stimulus feature information remaps. However, our results suggest that measuring remapping with fMRI may depend strongly on the details of the experimental paradigm. The apparent fragility of remapping as measured by fMRI, together with recent findings in other modalities (Churan et al., 2011; Zirnsak et al., 2011, 2014), suggest that remapping may not be as robust or general a phenomenon as has previously been supposed.

To provide relevant information for researchers considering studying remapping using fMRI, we highlight the differences between our study and the original fMRI studies of remapping and discuss potential reasons for the failure of the phenomenon to generalize to our paradigm. We also suggest directions for future research on the topic.

## Materials and Methods

The following methods are general to the three experiments in this study. Specific variations for each experiment are presented below:

### Subjects

Twenty subjects (12 females; mean age 26.1 years, age range 20–37 years) participated in at least one experiment; several subjects participated in multiple experiments. All subjects were neurologically intact with normal or corrected-to-normal vision. Informed consent was obtained for all subjects, and the study protocols were approved by the Massachusetts Institute of Technology Committee On the Use of Humans as Experimental Subjects.

### Stimuli and Task

Our focus in this experiment was to test whether feature-specific response patterns propagate to un-stimulated cortical regions following saccades. Thus to maximize signal we created a stimulus set consisting of two categories of images that differ in many features known to influence fMRI responses: faces and houses. Faces and houses differ in spatial frequency (Rajimehr et al., 2011), real-world size (Konkle and Oliva, 2012), semantic category (Schwarzlose et al., 2008), as well as the behaviors associated with each category. We reasoned that the feature differences between these categories of stimuli were likely to elicit differentiable response patterns in many visual regions, and that this feature information could be measured using multi-voxel pattern analysis (see “*Multivariate fMRI Analyses*” Section below).

**Figure 1:**
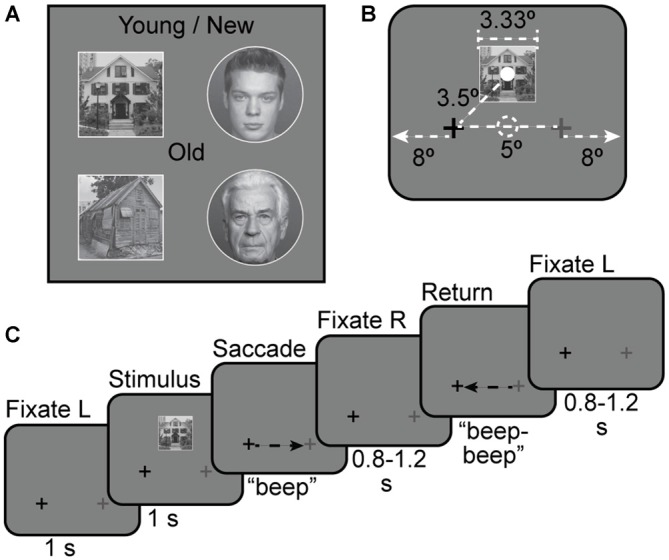
**Stimuli. (A)** Four examples of face and house stimuli used in the experiment. Young/new vs. Old distinction was the basis of the task in Experiments 2 and 3. **(B)** Stimulus size and location with respect to fixation points. The solid white dot indicates the center of the stimulus and the dashed white dot indicates the center of the screen; neither dot was shown during the experiment. Full screen size is not shown to scale. The screen extended for 8° out from either side of the fixation crosses. **(C)** Stimulus timing for a house + saccade left-to-right trial.

Our stimulus set consisted of house images collected from the Internet and neutral face images chosen from the FACES database hosted by Max Plank Institute for Human Development^1^. To further increase the low-level feature differences between the two classes of stimuli, we inserted each face into a circular white frame and each house into a square white frame (Figure 1A). To verify that the images in the face and house categories differed in low-level features, we parameterized each stimulus image with a Gabor wavelet feature space that quantifies local spatial frequency and orientation (Kay et al., 2008; Nishimoto et al., 2011). We computed the similarity between each pair of images in the Gabor wavelet feature space, and performed multi-dimensional scaling on the resulting similarity matrix. We found that the faces and houses each clustered together, and were readily separable from each other. This analysis shows that the face and house stimuli differ in features associated with responses in early visual cortex (Kay et al., 2008; Nishimoto et al., 2011). (See Supplementary Material for details).

Stimuli were presented using Psychtoolbox (Brainard, 1997; Kleiner et al., 2007) for Matlab (The Mathworks, Inc., Natick, MA, USA) and displayed with an LCD projector onto a screen mounted in the rear of the scanner bore. Subjects viewed the screen from a distance of 120 cm via a mirror attached to the head coil (maximal field of view (FOV): 21°).

Prior studies of remapping presented stimuli at approximately 8° in the periphery, but stimuli in the far periphery yield small estimates of feature information (Schwarzlose et al., 2008; Kravitz et al., 2010). In pilot experiments (data not shown) we found that estimates of feature information were higher for stimuli centered within 4° of the fovea. We chose to prioritize reliable feature information over exact replication of past work, in order to increase the number of regions that met our multivariate criteria for detecting feature remapping (see “*Signal Quality Criteria*” Section below).

Following previous studies of remapping in fMRI, we employed an event-related design (Merriam et al., 2003, 2007). For all experiments, subjects were instructed to maintain fixation on the darker of two crosses, located 2.5° to the left or right of screen center. See Figures 1B,C for an illustration of stimulus arrangement and trial timing. In each trial, a stimulus image was presented continuously for 1 s. Stimuli were presented on a neutral gray background. Stimulus images were 3.33° in diameter (for faces) and 3.33 × 3.33° square (for houses), and were displayed 2.5° above the screen center. This placed the stimuli 2.5° above and either 2.5° left or 2.5° right of fixation. Image-center-to-fixation distance was approximately 3.5°. On some trials, subjects were cued to move their eyes to the lighter cross by an auditory tone (“beep”). A second rapidly repeated tone (“beep-beep”) cued subjects to move their eyes back to the original fixation cross 1 ± 0.2 s later. For some trials there was no tone and subjects maintained fixation on the darker cross for the duration of the trial. For other trials no stimulus appeared and subjects made saccades as cued. The next trial began after a variable post-saccade interval of 0.8–1.2 s after the second tone. Each trial, from initial fixation to the end of the post-saccade interval, was 4 s long (Figure 1C). Additional gaps of 2 or 4 s were added between some trials to allow for timing jitter and better estimation of hemodynamic responses. The number of trials per condition, the distribution of trials per run, and the counterbalancing of conditions across subjects varied by experiment, and are discussed below.

For all trials, saccades were horizontal. We chose horizontal and not vertical saccades for both practical and theoretical reasons. Practically, adding a second saccade direction would have doubled the already large number of trial types, and would have created a further discrepancy with the paradigm used in previous work (Merriam et al., 2003, 2007). Theoretically, many regions that we wished to investigate have large receptive fields. Thus, different vertical positions for stimuli in the same hemisphere (with or without saccades) are likely to elicit nearly equal responses in these regions, which would make it difficult to distinguish remapping from stimulus-driven responses.

In addition to maintaining fixation and moving their eyes when cued, subjects also performed a detection task to keep their attention on the stimuli. In Experiment 1, subjects pressed a button whenever a red dot appeared superimposed on the stimulus. In Experiments 2 and 3, subjects pressed a button whenever a target image appeared. The target images were faces of older adults or old-looking houses; the standard stimuli were young adult faces or modern, well-maintained houses. Target events occurred rarely (4 per 64-trial run). Target-present and false alarm trials were removed from all subsequent analyses. Subjects were trained on all trial types with eye-tracking feedback before the scan, with the exception of the six naïve subjects of Experiment 3, who were trained only on the Saccade Only trials in advance. Subjects performed the detection task highly accurately—mean percent correct across subjects was 98.9%, 99.0%, and 96.8% in each of the three experiments.

### Trial Types vs. Stimulus Conditions

**Figure 2:**
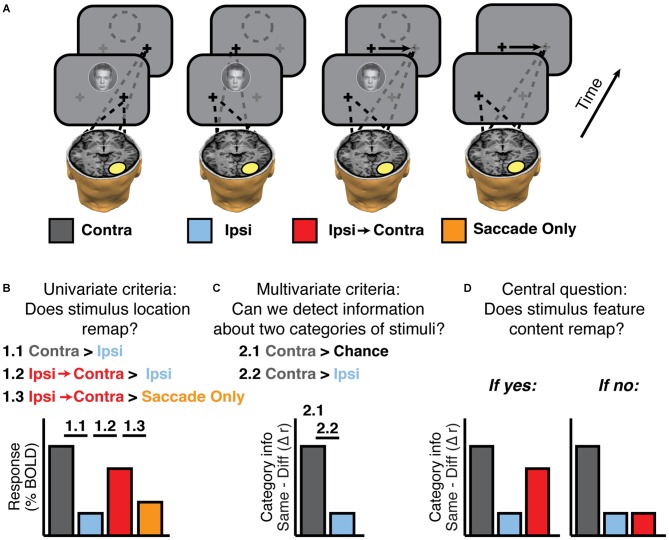
**Experimental logic. (A)** Experimental conditions, defined according to an example ROI in the brain (yellow oval). Fixation crosses and dashes lines indicate gaze position during and after stimulus presentation. The empty dashed circle indicates the memory trace of a stimulus. Neither the circle nor the arrows indicating saccades were actually presented; they are shown for display purposes only. **(B)** Univariate criteria to establish that the experiment has sufficient signal to find feature remapping if it exists. Activity in single voxels should show (1.1) greater responses to contralateral (Contra) than ipsilateral (Ipsi) stimulation, (1.2) greater responses to ipsilateral stimulation when followed by a saccade that brings the stimulus trace into the contralateral field (Ipsi-to-Contra), compared to Ipsi, and (1.3) greater responses to Ipsi-to-Contra than to a saccade with no stimulus (Saccade Only). **(C)** Multivariate checks to establish that the experiment has sufficient signal to find feature remapping. Activity in voxel patterns on the contralateral side should contain a significant amount of information (2.1), and more feature information on the contralateral side than the ipsilateral side (2.2). **(D)** Hypothetical outcomes of the multivariate analysis, either showing feature remapping or not.

For clarity, it is useful to make a distinction between *trial types* and *experimental conditions*. We use *trial types* to describe the locations of the stimuli relative to the center of the screen. Thus Figure 2A shows 4 out of 10 possible trial types: face + fixate right, face + fixate left, face + saccade left-to-right, no stimulus + saccade left-to-right. The six trial types not shown are face + saccade right-to-left, no stimulus + saccade right-to-left, and house + each of the eye positions described for faces. We assigned these 10 trial types to 10 *experimental conditions* defined by whether the stimulus was contralateral or ipsilateral to the voxels or regions being analyzed. For example, in Figure 2, the experimental conditions defined with respect to the highlighted region in the right hemisphere are: Contra, Ipsi, Ipsi-to-Contra, and Saccade Only. Note that the contra/ipsi labels would be reversed for regions of interest (ROIs) in the left hemisphere. Defining experimental conditions in terms of contralateral and ipsilateral position with respect to brain areas allows computation of aggregate statistics for ROIs across both hemispheres.

Note, that this paradigm requires a large number of conditions. In order to present enough trials per condition to make reliable estimates of feature information and still scan each subject in a single 2-h session, we chose to exclude the Saccade Only conditions from the first experiment and include them in follow-up experiments. Based on previous work (Merriam et al., 2003, 2007), we anticipated that the “remapped” response (Ipsi-to-Contra condition) would be greater than the Saccade Only control response, and our main focus of Experiment 1 was to test whether stimulus feature information could be detected in that “remapped” response. For Experiments 2 and 3 we included the Saccade Only controls, but excluded some stimulus presentations on one side of the visual field to allow us sufficient power for each condition.

### Eye Tracking

Eye position was monitored using a modified ISCAN eye tracking system (ISCAN, Inc., Burlington, MA, USA) for Experiments 1, 2 and the first half of Experiment 3, and an EyeLink eye tracking system (SR Research Ltd., Mississauga, Ontario, ON, Canada) for the second half of Experiment 3. For both eye tracking systems, the camera and infrared source were placed directly in front of the bottom of the rear screen. Pupil and corneal reflection (CR) were recorded at 120 Hz and analyzed offline to ensure accurate fixation performance. The eye tracker was calibrated at the beginning of the session and repeated between runs if necessary and as time permitted. When the eye-tracker signal in the scanner was too noisy to achieve reliable calibration, the experimenter monitored eye position via the live video from the eye tracking camera. All subjects were expert subjects with substantial practice in eye-tracking tasks outside the scanner, and were trained on the task with eye tracking feedback before the scan.

To remove calibration drift over the course of each scan, the median eye position in the epoch from 600 to 100 ms before stimulus onset in each trial was subtracted from the data for that trial. The expected trial start fixation position (for example, [+2.5°, 0°]) was then added back to the eye position data. For trials with eye movements, the median eye position in the epoch from 1750 to 2250 ms after stimulus offset was also computed, and the data were then re-scaled using the difference between median eye positions in the post-saccade fixation and the pre-stimulus fixation intervals. The epochs used for eye position normalization were outside the period of interest for each trial (before stimulus onset and after saccade completion).

Trials were automatically discarded if eye position recording lapsed for more than 350 ms or if more than 10% of the eye positions recorded for a given trial were more than 10° from fixation. For the remaining trials, human raters reviewed the normalized eye data for each subject and discarded trials with excessive noise, missing data (due to blinks or eye tracker malfunction), any saccades other than the cued saccades, and eye drift away from fixation. The human raters were naïve as to the purpose of the experiment and the resulting data. Many trials were discarded, most commonly due to noise or missing data. Comparatively few trials were discarded based on poor eye behavior (for example, breaking fixation, mistimed saccades, or saccades to the image). Thus the high discard rates reflect noisy data and a conservative standard for keeping eye trials.

All trials retained by the raters were used to compute mean saccade onset times for each experiment. To automatically label saccades, we first computed a measure of eye position dispersion for all data points within a sliding window 100 ms wide. Our dispersion measure was the sum of the *x* and *y* eye position ranges (in degrees). If the dispersion of the eye position data was greater than 3° over a given 100 ms window, the measurement at the center of the window was labeled as a part of a saccade. Saccadic latency was computed as the time from saccade cue onset to the first time point labeled as a saccade.

To assure that poor eye behavior did not affect the fMRI results, data from trials meeting our conservative inclusion criteria were re-analyzed. For this analysis, subjects were excluded entirely if more than 75% of trials were discarded for a given experimental condition, or if more than 50% of all trials were discarded for that subject.

### fMRI Parameters

Magnetic resonance imaging (MRI) scanning was carried out with a Siemens Trio 3-T scanner using a 32-channel receiver array head coil. Functional data were acquired with a T2*-weighted gradient-echo sequence (repetition time (TR) = 2000 ms, echo time (TE) = 30 ms, flip angle = 90°, FOV = 192 mm × 192 mm, voxel size = 1.5 × 1.5 × 2 mm, inter-slice gap = 0.4 mm). Parallel imaging (Siemens iPAT with an acceleration factor of 2) was used, and 28 approximately axial slices were collected angled ~30° forward (perpendicular to the calcarine sulcus), to maximize high-resolution coverage of occipital, parietal, and posterior temporal cortices. The same fMRI protocol was used for the main experiment and the functional localizer scans.

### fMRI Preprocessing

fMRI data were temporally interpolated to align each slice with the first slice acquired, motion corrected (with trilinear-sinc interpolation), and temporally high-pass filtered to remove low-frequency drift (kernel = 0.01 Hz). No spatial smoothing was performed on the data beyond the interpolation required for motion correction and cross-run alignment. Preprocessing was carried out using a combination of Brain Voyager QX version 2.2 (Brain Innovation, Mastricht, Netherlands; Goebel et al., 2006) and the fMRI Software Library (FSL; Smith et al., 2004; Jenkinson et al., 2012). All univariate and multivariate analyses in the main experiments were conducted in native functional space using custom Matlab code.

### Functional Localizers

All functional ROIs were defined based on three different localizers collected independently of the main experiment. The threshold for each localizer contrast was set at *p* < 0.05, corrected for False Detection Rate [FDR], Benjamini and Yekutieli, 2001). All category-selective regions as well as Lateral Occipital cortex (LO) were defined based on specific functional contrasts combined with constraints from anatomy and relative location (Weiner and Grill-Spector, 2011).

To define the FFA, the OFA, the OPA, and the PPA we used a localizer with 20 blocks of static images of faces and houses. Within each block, 24 images were presented for 500 ms each. FFA and OFA were defined by a contrast of Faces > Houses (Golomb et al., 2011). FFA was constrained to be on the fusiform gyrus, and OFA was constrained to be on the inferior occipital gyrus (IOG; Weiner et al., 2014). PPA and OPA were defined by a contrast of Houses > Faces. PPA was constrained to be medial to the mid-fusiform gyrus, and OPA was constrained to be lateral to the base of the intraparietal sulcus and near (but not necessarily in) the transverse occipital sulcus (Dilks et al., 2013).

For three subjects, face/house localizers were not collected due to time limitations. For one of these subjects (Experiment 1 subject 7), we used data from a pilot version of the main experiment to compute a contrast of all face conditions > all house conditions. We used this contrast to define face- and house-selective regions (we kept voxels that showed a statistical difference of *p* < 0.05, uncorrected).

To define Lateral Occipital cortex (LO), we used a localizer consisting of blocks of images of static objects and scrambled versions of the same objects (Malach et al., 1995; Grill-Spector et al., 1999). LO was defined by a contrast of Objects > Scrambled objects, and further constrained to the area anterior to V4, between OPA dorsally and V4 or OFA ventrally (Hansen et al., 2007).

V1–V4 were defined using a localizer consisting of alternating flashing vertical and horizontal double-wedge (bowtie) stimuli (Slotnick and Yantis, 2003; Qiu et al., 2006). The vertical and horizontal meridia were demarcated based on a contrast between blocks of vertical and horizontal stimuli. For each ROI, a region between the relevant meridia was selected that showed a significant contrast between contralateral and ipsilateral image conditions in the object/scramble localizer scans. This procedure assured that the eccentricity of each ROI matched the eccentricity of the stimuli in the main experiment.

All images in the localizers (except meridian-mapping bowties) were presented in the same retinal locations the stimuli in the main experiment. For one subject in Experiment 3, the images in both the main experiment and the localizers were presented slightly closer to fixation and smaller than for the other subjects due to a code error.

Functional data were co-registered to a high-resolution 3D multi-echo magnetization prepared rapid gradient echo (ME-MP-RAGE) anatomical scan collected in the same session. For each subject, functional localizer data was projected onto a Talairach-space cortical surface mesh created from the subject’s anatomical scan. ROIs were defined on this surface and transformed back to the native functional space.

### Univariate fMRI Analyses

All runs were analyzed with a standard general linear model, with 10 finite impulse response (FIR) predictors for each condition and one predictor for each run. Weights for each predictor for each voxel were fit using ordinary least squares regression.

We obtained a single activation value per voxel per condition by averaging the weights of the FIR predictors near the peak of the hemodynamic response function (HRF) for each condition. To find the peak of the HRF per condition, we averaged the FIR weights across voxels in each ROI and identified the FIR predictor for each condition with the maximum average weight. We then used *t* tests across all voxels in each ROI to compare the FIR predictors within 4 s (2 TRs) of the maximum to the maximum FIR predictor. We averaged all FIR weights that were not significantly different from the maximum (*p* > 0.05). This procedure was performed separately for each condition, and selected 1–4 FIR predictors comprising the HRF peak for each ROI for each condition. The same time points per condition were averaged for all voxels in a given ROI. This procedure allowed for variation in HRF shape across ROIs and subjects.

Activation values for each condition were converted to units of percent signal change by dividing the average weight for each condition by the average signal intensity across runs. At this stage, the trial types were assigned to ipsilateral/contralateral experimental conditions according to the position of the stimulus and the direction of the eye movement with respect to each region of interest (as described above). For example, the face + fixate right trial type (in which a face appeared to the left of a fixation cross on the right) was assigned to the Face-Ipsi condition for voxels in the left hemisphere and the Face-Contra condition for voxels in the right hemisphere. For all univariate analyses, percent signal change values for each condition were averaged across hemispheres after this conversion.

In Experiments 1 and 3, activation values for each condition were compared across subjects using paired *t* tests. In Experiment 2, due to the limited number of subjects, significance of the difference between conditions was assessed within each individual subject using the following procedure. Activation values for each condition were estimated 300 times, each time using 80% of the available data (selected with replacement). Using these 300 bootstrapped estimates, a 95% confidence interval was computed for each relevant difference between conditions. If the 95% confidence interval for a given difference (for a particular subject in a particular ROI) did not contain zero, that difference was considered significant.

### Multivariate fMRI Analyses

We used multi-voxel pattern correlation (Haxby et al., 2001; Cox and Savoy, 2003) to determine whether the pattern of activity in each ROI could distinguish faces from houses. Distinguishable patterns in a given area imply that the area contains information about the features that differ between the face and house stimuli. Thus, we refer to the multivariate measure of pattern separability (which we define below) as *feature information*.

For all analyses, each subject was first analyzed independently, and measures of feature information per subject per condition were averaged at the end. To determine whether a given region contained information about the distinction between the features in our two categories of stimuli, we first split the data for each subject in half *n* different ways. The number of splits (*n*) varied by experiment, due to different numbers of trials per experiment. We then estimated response amplitudes to each condition for each voxel in each half of the data as described in *Univariate fMRI analyses*. This resulted in a vector of estimated voxel responses for each condition, for each ROI, for each half of the data, for each way to split the data in half. We normalized the responses by subtracting the mean response across conditions for each voxel from the response to each separate condition for that voxel, as in Haxby et al. (2001). This was done independently for each half of the data, and for each split. For each split, we generated a correlation matrix by correlating the voxel response vectors for all conditions in one half of the data with those in the other half. Correlations were Fischer *z*-scored and then averaged over the different splits. We used a difference of *z*-scored correlations (Δ*r*) as a measure of feature information:

(1) $$\Delta r\;=\;\frac{Z\left(r_{fA,fB}\right)+Z\left(r_{hA,hB}\right)}{2}-\frac{Z\left(r_{fA,hB}\right)+Z\left(r_{hA,fB}\right)}{2}$$

where, *f* and *h* refer to faces and houses, and *A* and *B* refer to the first and second half of the data set, respectively. Thus *r_fA, hB_* is the correlation between the response pattern to faces in the first half of the data set and the response pattern to houses in the second half of the data set. Δ*r* will be greater than zero if multi-voxel patterns are more similar within categories than across categories. Thus, the values of Δ*r* provide an estimate of feature information: how much information is conveyed in multi-voxel response patterns about the feature differences between stimulus categories. We computed Δ*r* independently for each eye movement condition (Contra, Ipsi, and Ipsi-to-Contra).

In Experiments 1 and 3, we compared measures of feature information in each ROI across subjects using *t* tests. In Experiment 2, we used a nonparametric method to determine whether feature information (Δ*r*) was significantly different from zero for each ROI in each individual subject. First, we split the data in half 100 different ways, and computed estimates of the correlations between each pair of conditions for each split. We then computed differences of correlations between different conditions for each split. This yielded 100 bootstrapped Δ*r* values for each ROI. Finally, we computed a 95% confidence interval for Δ*r* for each condition in each ROI by resampling different combinations of the 100 estimates of Δ*r* for each condition and ROI. If the 95% confidence interval did not contain zero for a given condition and ROI, we concluded that feature information was reliably different from zero.

### Signal Quality Criteria

For each region in this experiment, we investigated remapping of stimulus *location* (using univariate mean response measures) and remapping of stimulus *features* (using the MVPA feature information (Δ*r*) measure). To establish that we had sufficient signal to detect feature remapping if it did exist, we set two types of criteria for signal quality in each region that we investigated.

First, we tested whether each region showed univariate stimulus *location* remapping as in Merriam et al. (2003, 2007). That is, we identified regions that responded more strongly to contralateral stimuli than ipsilateral stimuli (Figure 2B, 1.1) and then tested whether each region responded more in the Ipsi-to-Contra condition than in the Ipsi condition (Figure 2B, 1.2). These were the only criteria used for evaluating location remapping in Experiment 1. In Experiments 2–3, we set the additional criterion that the response in the Ipsi-to-Contra condition must be greater than the response in the Saccade Only condition (Figure 2B, 1.3).

Second, we tested whether we had enough signal to measure remapping of stimulus *feature* information in each region. We tested whether there was significant feature information in the Contra condition (i.e., whether we could distinguish our two categories of stimuli (faces in circles and houses in squares) based on patterns of voxel responses when the stimuli were presented contralaterally (Figure 2B, 2.1), and then tested whether there was greater feature information in the Contra condition than in the Ipsi condition (Figure 2B, 2.2).

Finally, with these criteria in place, we tested whether each candidate region exhibited *remapped* feature information. In other words, was there more feature information in the Ipsi-to-Contra condition than in the Ipsi condition (Figure 2D, 2.2)?

It is worth noting that there are many reasons we might fail to measure an increase in feature information following a saccade. Also, as with any null result, a failure to find feature remapping does not necessarily mean that it does not exist. However, the absence of feature remapping becomes more conspicuous if a given region meets all of these signal quality criteria described above.

## Experiment 1

In Experiment 1 we conducted a test of feature remapping with eight trial types: faces and houses for fixate right, fixate left, saccade left-to-right, and saccade right-to-left. As noted above, we did not include the Saccade Only conditions in this initial experiment because we wanted to ensure sufficient statistical power for the main conditions of interest.

### Methods

Ten subjects (three females, mean age 26.5 y, age range 21–36 y) participated in Experiment 1. One subject was rejected for excessive head movements, resulting in nine usable subjects. Six of these subjects had already participated in pilot versions of the study. Subjects saw eight different trial types in each of 10 to 12 runs of scanning. Due to time constraints, one subject had two fewer runs than the others. Each run had 64 trials, including four target trials, for a total of ~90 trials per trial type after target and false alarm trials were removed. Trial types were pseudo-randomly intermixed with a constraint to minimize temporal correlations between condition onsets. The location of the initial fixation cross changed sides of the screen halfway through each run, so all conditions were presented in each run. Initial fixation position for the first half of the trials was balanced across different runs/subjects.

### Results

#### Eye Tracking

**Figure 3:**
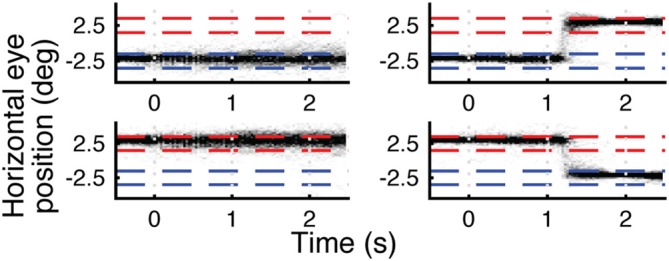
**Eye traces for horizontal (x) eye position for four different eye movement trial types for a single subject.** Top left is fixate left, top right is saccade left-to-right, bottom left is fixate right, bottom right is saccade right-to-left. Each plot is a density plot containing trials for both face and house stimuli. The darkness of each pixel reflects the number of trials (up to a max of 30) in which the subject’s eyes were focused on a given × location during each 30 ms window. Blue and red dashed lines indicate left and right fixation targets ±1°. Stimulus onset is at 0 s and offset is at 1 s. Eye behavior was accurate and consistent across trials.

Eye traces for one subject are shown in Figure 3. This subject had reliable eye tracking data signal quality, and the eye traces confirm successful execution of the fixation and saccade tasks. Across all subjects, 54% (3627/6720) of the trials were retained for eye tracking analysis. For trials requiring saccades, average latency (± standard deviation) from saccade cue to saccade onset was 272 ± 104 ms. This latency is well within the window for memory trace remapping (Duhamel et al., 1992), and comparable to saccadic latencies in previous reports of memory trace remapping in fMRI (Merriam et al., 2003, 2007). Analyzing fMRI data from only trials with verifiably good eye behavior gave highly similar results to those reported below. For details of this analysis and further analysis of eye tracking data (including eye data for Experiments 2 and 3), see Supplementary Materials.

#### Stimulus Location Remapping

**Figure 4:**
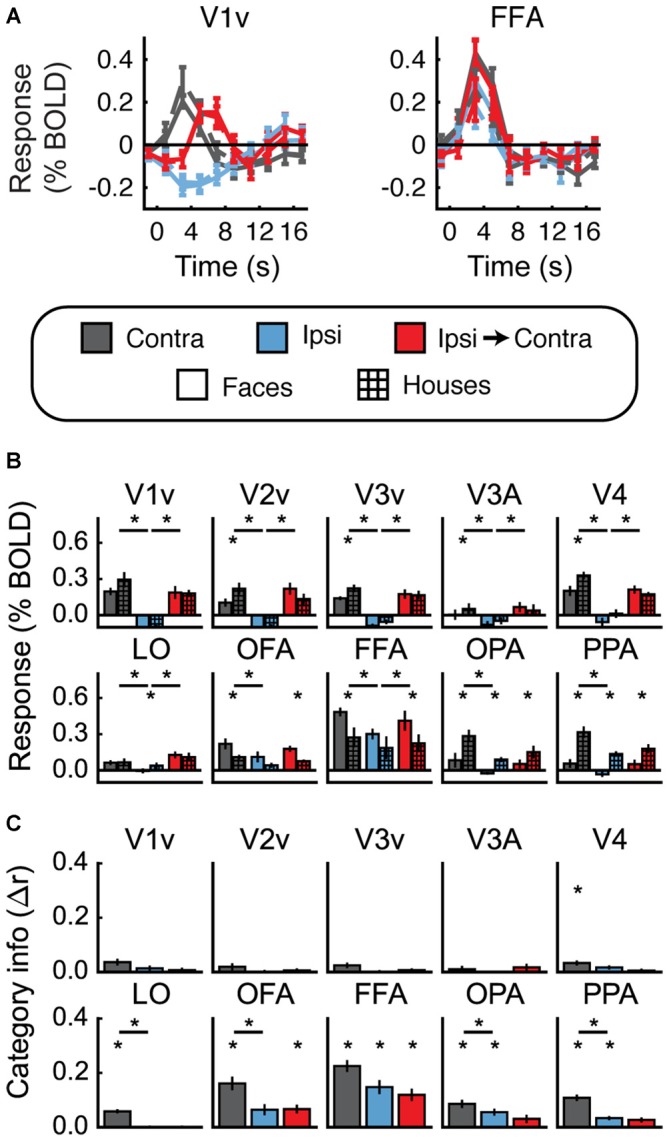
**Experiment 1 results. (A)** Estimated hemodynamic response function (HRF) functions for two representative ROIs (V1v and fusiform facearea (FFA)), according to the legend below. “v” Denotes the ventral portion of the ROI, which represents the upper visual field where the stimuli appeared. **(B)** Bar graphs for estimated response (in percent signal change) per condition per ROI. The lower level of asterisks indicate significant (*p* < 0.05) differences between face and house responses. Lines with asterisks above them denote significant (*p* < 0.05) differences between eye movement conditions (e.g., between Contra and Ipsi). V1v-V4, LO, and FFA all show a pattern of responses consistent with remapping of stimulus location. **(C)** Feature information (difference of *z*-scored Pearson correlations, Δ*r*) for each ROI. Asterisks directly above the bars indicate significant feature information for that condition (Δr > 0, *p* < 0.05). Asterisks between bars indicate significant differences in feature information (*p* < 0.05). No region shows a pattern of responses consistent with remapping of feature information (an increase in feature information in the Ipsi-Contra condition vs. the Ipsi condition).

Figures 4A,B shows the univariate results for Experiment 1. For each ROI, data are averaged across hemispheres. Both faces and houses activated all of the visual areas we defined, with OFA and FFA responding more to faces and OPA and PPA responding more to houses. Importantly, all of these areas also exhibited a contralateral preference; that is, significantly greater activity for Contra vs. Ipsi (all *t* > 2.6, *p* < 0.05).

Does this univariate activity remap? Consistent with Merriam et al. (2003, 2007), we observed larger responses in the Ipsi-to-Contra condition than in the Ipsi condition in several visual areas (for V1–V4, V3A, LO, and FFA, all *t* > 2.8, *p* < 0.05; see asterisks in Figure 4B). Each of these regions meets both of the criteria for univariate remapping that were tested in this experiment (Figure 2B, 1.1 and 1.2). Supplementary Table 1 provides statistics for all regions individually.

#### Feature Information Detection

Figure 4C shows the feature information results for Experiment 1. The patterns of voxel activity in early visual cortex did not distinguish between our face and house images, despite our attempts to incorporate low-level differences into the stimuli. However, in V4, LO, OFA, FFA, OPA, and PPA, we observed significant amounts of feature information for faces vs. houses (all *t* > 2.3, *p* < 0.05). LO, OFA, OPA, and PPA had greater amounts of feature information in contralateral vs. ipsilateral hemispheres (all *t* > 2.6, *p* < 0.05).

#### Feature Information Remapping

To address the key question of whether stimulus feature information remaps, we tested whether feature information was greater in the Ipsi-to-Contra condition than in the Ipsi condition. Despite the increase in univariate response in many regions (Figure 4B), we observed no analogous increase in feature information in any region tested (Figure 4C, Supplementary Table 1).

### Discussion

In summary, we found tentative evidence for stimulus location remapping in areas V1–V4, V3A, LO, and FFA, and sufficient power to detect feature information in V4, LO, OFA, FFA, OPA, and PPA. Of these regions, only LO met all the signal quality criteria defined in the Methods. (FFA and V4 met most of these criteria, but did not show more feature information with contralateral stimulus presentation than with ipsilateral stimulus presentation.) For our key question of interest, we found no evidence for automatic remapping of stimulus feature information, in LO or any other region.

However, any conclusion based on the data presented so far is qualified by two important weaknesses of Experiment 1. First, fMRI is particularly susceptible to Type II error: it is easy to miss real effects because of poor signal. Any regions that pass our signal quality criteria should have the statistical power to detect feature remapping if it exists, but only one ROI met all our criteria (LO), and feature information in LO was relatively low compared to other regions. More trials per condition would likely provide better signal to help with this issue. Second, we did not include Saccade Only control conditions in Experiment 1. Firmly establishing location remapping requires a test of whether univariate responses to ipsilateral stimuli followed by a saccade are greater than responses to saccades alone (Figure 2B, 1.3), as was done in Merriam et al. (2003, 2007). Experiment 2 addresses both of these issues.

## Experiment 2

Experiment 2 used the same general design as Experiment 1, with a few modifications. First, to fully establish that we had measured location remapping, we added two Saccade Only control conditions, one in each direction: Contra-to-Ipsi Saccade Only and Ipsi-to-Contra Saccade Only. Second, to increase the number of trials per condition for feature information analyses, we restricted the stimuli to one side of the visual field per subject.

We also changed the task to encourage subjects to attend to the features—rather than merely the location—of each stimulus image. Subjects were instructed to press a button whenever they saw an occasional target image of an old face or house, compared to the more frequent non-target images of young faces and modern houses (Figure 1A). This task is orthogonal to the feature information analysis and did not require sustained attention to or memory of each image after stimulus presentation. This is in keeping with our goal to study automatic stimulus feature remapping rather than mechanisms of attention shifts, which we view as a separate question.

We made one other change to the timing of the saccade cues, which resulted in only a minor change in the latency from image offset to saccade onset (see supplemental materials: eye tracking results).

### Methods

Four subjects (two females, mean age 26.0 years, age range 21–32 years) participated in this experiment. Three of the subjects had also participated in Experiment 1. Two subjects saw the five conditions with the initial fixation to the right of screen center, and two saw the five conditions with the initial fixation position to the left of screen center, in each case over 12–13 runs of scanning. Each run had 64 trials, including 4 target trials, for a total of ~156 usable trials per condition after target/false alarm trials were removed. Conditions were pseudo-randomly intermixed with a constraint to minimize correlations between condition onsets. This resulted in ~70% more trials per condition than Experiment 1, at the cost of having only half of each subject’s brain exposed to each condition. (For a given hemisphere, responses were only measured for either Contra, Contra-to-Ipsi, and Contra-to-Ipsi Saccade Only conditions, or for Ipsi, Ipsi-to-Contra, and Ipsi-to-Contra Saccade Only conditions).

### Results

#### Stimulus Location Remapping

**Figure 5:**
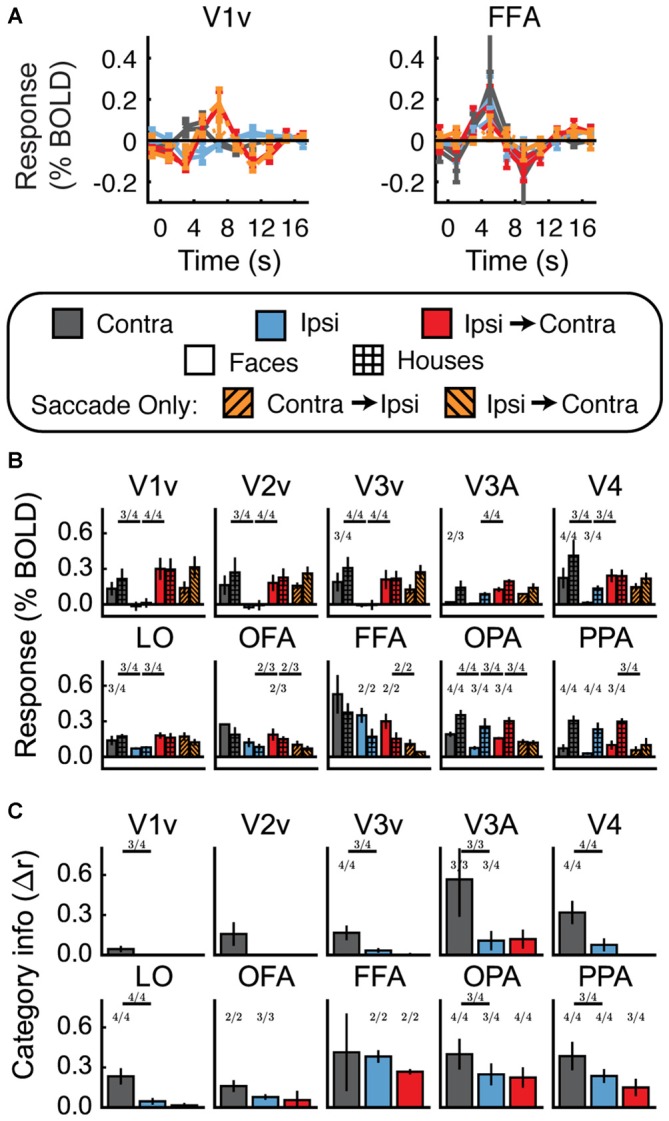
**Experiment 2 results. (A)** Estimated HRF functions for two representative ROIs (V1v and FFA). “v” Denotes the ventral portion of the ROI, which represents the upper visual field where the stimuli appeared. **(B)** Bar graphs for estimated response (in percent signal change) per condition per ROI. Fractions indicate the number of subjects for whom each difference was reliable. Fractions above lines denote the number of subjects who showed reliable differences between eye movement conditions (e.g., between Contra and Ipsi). In V1v-V4, V3A, LO, OFA, and OPA, a majority of subjects show larger responses in Ipsi-to-Contra condition than in the Ipsi condition. However, only in OFA and OPA are the Ipsi-to-Contra responses larger than Saccade Only responses in a majority of subjects. Thus, only OFA and OPA show patterns of responses that are consistent with remapping of stimulus location. **(C)** Category information (difference of *z*-scored Pearson correlations, Δ*r*) for each ROI. Fractions indicate the number of subjects for whom category information (or a difference in category information) was reliably greater than zero. No region shows a pattern of responses consistent with remapping of feature information (an increase in category information in the Ipsi-Contra condition vs. the Ipsi condition).

Univariate results are shown in Figures 5A,B (see Supplementary Table 2 for statistics by subject and ROI). As in Experiment 1, most areas exhibited a contralateral preference, and the category-selective areas exhibited stronger responses for their respective preferred categories. A majority of subjects (at least 3/4, or 2/3 in OFA) showed partial evidence for stimulus location remapping in V1–V4, LO, OFA, and OPA, with reliably larger responses in the Ipsi-to-Contra condition than in the Ipsi condition (a 95% confidence interval on the difference between the two conditions did not contain zero). However, responses to the Saccade Only condition were also substantial in all ROIs. Of the ROIs exhibiting tentative evidence for location remapping, only in OFA and OPA were responses in the Ipsi-to-Contra condition larger than responses in the Saccade Only condition. The only region that met all the criteria we established for location remapping was OPA, and in OPA only two of the four subjects met all the criteria.

#### Feature Information Detection

Multivariate results for Experiment 2 are shown in Figure 5C and Supplementary Table 2. In all ROIs, we observed more feature information in the contralateral responses than we observed in Experiment 1 (note the scale of the axes in Figure 5C vs. Figure 4C), likely because of the increased number of trials per condition and the task requiring more attention to stimulus features. We found feature information in the contralateral responses in a majority of subjects (at least 3/4) in V3, V3A, V4, LO, OFA, OPA, and PPA (95% confidence intervals for feature information did not contain zero—see Supplementary Table 2). Moreover, feature information was significantly greater in the Contra condition than in the Ipsi condition in V1, V3, V3A, V4, LO, OPA, and PPA. (The reason that we did not find feature information in FFA is likely that we could only localize FFA in three of the four subjects. Furthermore, in two of those three subjects, we could only localize FFA in the right hemisphere, and one of those subjects did not see stimuli contralateral to right FFA at all).

#### Feature Information Remapping

Despite the reliable presence of feature information in response to contralateral stimuli, feature information in the Ipsi-to-Contra condition was not reliably higher than feature information in the Ipsi condition in any ROIs. Thus, neither Experiment 1 nor Experiment 2 provided any evidence for remapping of stimulus feature information.

### Discussion

Experiment 2 replicated most of the findings of Experiment 1, and the greater power to detect feature information makes the lack of stimulus feature remapping even more salient. However—surprisingly, given prior results (Nakamura and Colby, 2000, 2002; Merriam et al., 2003, 2007)—we observed statistically indistinguishable response magnitudes in the Ipsi-to-Contra and Saccade Only conditions. Large responses in the Saccade Only condition raise the possibility that the responses in the Ipsi-to-Contra condition were exclusively due to the presence of a saccade. Our results do not necessarily mean that there was no remapping of stimulus location. It is possible that responses due to remapping and responses due to saccades do not sum linearly, so it is impossible to say: (1) whether a real effect of location remapping may have been masked in our data by a large response to saccades alone; or (2) whether there was no remapped response at all. Given this ambiguity, it is difficult to draw any firm conclusions about stimulus feature remapping based on the data presented so far.

Why were the Saccade Only responses so large in the current experiment? One possibility is that the luminance difference between the center of the screen and the periphery contributed to the large Saccade Only responses. The receptive fields of neurons in all ROIs were always centered on some part of the gray projection screen. However, the part of the screen that stimulated the ipsilateral ROIs was closer to the visual periphery, and the periphery may have been dimmer overall due to the lack of illumination from the dark area outside the projection screen. Thus, in the Ipsi-to-Contra condition and Ipsi-to-Contra Saccade Only condition, the subjects’ eye movements may have brought a slightly brighter part of the screen into the receptive fields of the neurons in each ROI. Consistent with this possibility, responses in the Ipsi-to-Contra Saccade Only condition were slightly larger than responses in the Contra-to-Ipsi Saccade Only condition for most ROIs in most subjects (see Supplementary Table 2).

**Figure 6:**
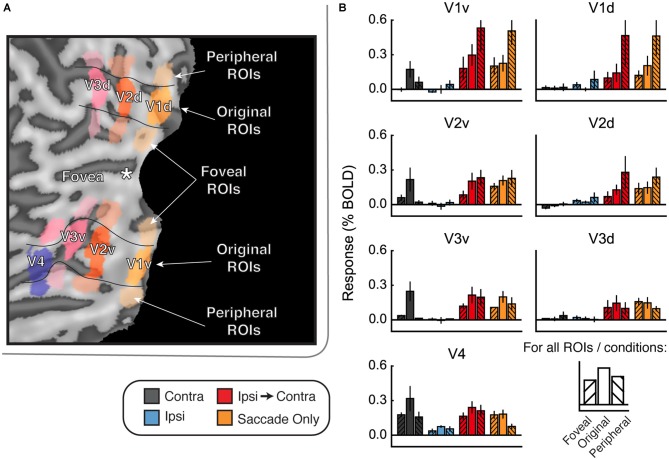
**Foveal/peripheral ROI analysis for Experiment 2. (A)** Locations of each ROI for one example subject. **(B)** Responses within the foveal, original, and peripheral ROIs. Responses to faces and houses are averaged together in each bar. In the Contra condition, responses are largest in the original ventral ROIs. In the Ipsi-to-Contra condition and the Saccade Only condition, responses are largest in the most peripheral ROIs. This suggests that something in the visual periphery affected the responses more strongly than the stimulus, and casts doubt on whether the Ipsi-to-Contra responses should be interpreted as remapping.

This difference between saccades in different directions suggests that illumination or some other factor may have affected the responses we measured. If this were the case, then regions of the cortex that represent the visual periphery should respond more strongly than the original ROIs in the Ipsi-to-Contra and Saccade Only conditions. To explore this possibility, we defined ROIs in both ventral and dorsal portions of V1–V4 that were more foveal and more peripheral than our original ROIs (Figure 6A). We used these new ROIs to explore how much the responses in the Ipsi-to-Contra and Saccade Only conditions depended on eccentricity of the receptive fields for each ROI.

Figure 6B shows responses in the foveal and peripheral ROIs compared to the original ROIs. In the Contra condition, responses are higher in the central ROIs than in the peripheral or foveal ROIs. In the dorsal ROIs, no response to the Contra condition is apparent. This analysis shows that our ROIs did, in fact, encompass the location in the brain that responded to the visual location of the stimulus. In both the Ipsi-to-Contra condition and the Saccade Only condition, however, responses were not limited to the same patch of cortex. In all regions except V4, responses in the peripheral ROIs were equal to or larger than responses in the central ROIs. This suggests that some factor in the periphery strongly influenced responses in our paradigm.

A final possibility that could contribute to increased responses in the Saccade Only condition relates to stimulus expectation. Even though stimuli were never actually presented on the Saccade Only trials, these trials were randomly intermixed with stimulus trials, and subjects may have established an ongoing expectation or memory of stimuli appearing at that particular location on other trials. Umeno and Goldberg (2001) demonstrated that some neurons in macaque FEF will begin to respond to a saccade alone if saccade trials are interleaved with trials in which a stimulus appears in a predictable location—even if they did not respond to a saccade initially. In the original article using the fMRI remapping paradigm, Merriam et al. (2003) chose to present their conditions in a fixed order in the majority of their data collection to avoid this problem.

We designed Experiment 3 to address these potential confounds with illumination and stimulus expectation, both of which could have caused an increased Saccade Only response that obscured real location remapping.

## Experiment 3

In order to more closely replicate previous work, and to reduce or eliminate two potential causes of large Saccade Only condition responses, we made two changes in the experimental design for Experiment 3. To reduce the effect of global illumination on the blood-oxygen-level dependent (BOLD) responses, we eliminated ambient light sources from the scanning environment and darkened the screen background. To reduce the chance that subjects’ expectation of a stimulus would drive responses higher in the Saccade Only condition, we grouped our conditions together by run and presented all the Saccade Only trials together in the same runs. We also varied the order of presentation of the Saccade Only condition with respect to the other conditions. For half the subjects, the runs containing Saccade Only trials appeared before all other runs; for the other half, the Saccade Only condition was interleaved with the runs for other conditions.

We note that the grouping of conditions by run is not consistent with modern fMRI best practices. If conditions are not randomly interleaved, the odds increase that general alertness, cognitive expectation, or scanner-related artifacts could vary across conditions and confound the results. However, one goal of this experiment was to isolate the cause of the discrepancy between our work and past work, so we matched our stimulus presentation protocol to previous studies to see if results based on grouped conditions would match previously reported results more closely than the results of our initial interleaved design.

### Methods

Twelve subjects (nine females, mean age 25.8 years, age range 20–30 years) participated in Experiment 3. Three of the subjects had participated in one of the prior experiments. One subject was excluded for excessive drowsiness and consequently poor fixation behavior. Of the 11 remaining subjects, five were scanned in an additional session, to provide more data for the feature information analysis.

For Experiment 3 we included 7 of the 10 possible trial types. As in Experiment 2, each subject was only required to make eye movements in one direction. Thus, every subject saw faces and houses presented on both sides of the visual field without making a saccade, and also saw either: (1) left-to-right faces, left-to-right houses, and left-to-right saccade-only trials; or (2) right-to-left faces, right-to-left houses, and right-to-left Saccade-Only trials.

To match previous experimental designs (Merriam et al., 2003, 2007), we only showed one type of condition for each scanning run (though face and house trials were still intermixed). Half of the subjects saw Saccade Only runs interleaved with runs of the other conditions, with the order of scanning runs randomly assigned. The three subjects who had participated in previous experiments were all scanned in this version of the experiment. The other half of the subjects saw the Saccade Only condition presented first to further diminish the possibility that an expectation or memory of a stimulus would affect the BOLD responses across condition blocks. The six subjects for this version were all naïve to the intent of the experiment and had not seen any trials with stimuli in them before viewing the Saccade Only runs. For these subjects, task instructions were given in progressive sections before each type of run: before the experiment they were only given the instructions (and eye-tracker practice) for the Saccade Only task; instructions for the subsequent conditions were given while subjects were in the scanner after completion of the Saccade Only runs. All subjects performed the same detection task as in Experiment 2 (for trials with stimuli present).

12–18 runs were collected per subject for the first session. Each run had 48 trials, including 4 target trials, for a total of ~100 trials per condition after target and false alarm trials were removed. 12–15 more runs were collected for subjects who participated in a second session, resulting in ~200 trials per condition. For subjects with multiple runs, functional data were aligned to an anatomical scan collected in the same session and transformed to a reference frame defined based on the location of the subject’s anterior and posterior commissures (commonly referred to as the AC-PC reference frame) using a 6-parameter (rigid body) affine transformation.

The fMRI scanning environment was also modified to minimize background illumination by changing the stimulus background to black, turning off all lights in the scanner room, and draping blackout sheets over the observation window separating the scanner room from the control room to prevent outside light from entering the scanner room. The only light sources thus came from the projector itself. However, even the black background still produced a measurable amount of background illumination when the projector was on. To reduce this illumination as much as possible, we additionally placed a thick neutral-density filter in front of the entire projector, and further blocked extraneous illumination by placing an opaque black cardboard cutout over the projector blocking un-stimulated parts of the screen.

### Results and Discussion

#### Effect of Condition Order

We compared both versions of the experiment (interleaved order vs. Saccade Only first) to test whether expectation of the stimulus appearing affected responses in each condition. First, we estimated responses for each condition for subjects in the interleaved version, for whom blocks of Ipsi-to-Contra and Saccade Only trials were interleaved with blocks of other trial types. Then we estimated responses for each condition for subjects in the Saccade Only first version, who had seen all the Saccade Only trials in the first blocks and Ipsi-to-Contra trials in the latter blocks. We averaged responses to faces and houses to get a single response per eye movement condition per ROI, and performed a two-way analysis of variance (ANOVA) on the responses with within-subject factor Condition (Contra, Ipsi, Ipsi-to-Contra, and Saccade Only) and between-subject factor Order (interleaved and Saccade Only first). We found a main effect of Condition in all ROIs except the dorsal (un-stimulated) parts of V1, V2, and V3 (all *F*_(3,1)_ > 8.25; see Table 1 for statistics). We also found a main effect of Order in V1d, V2v, V2d, V3v, V3d, and V4 (all *F*_(3,1)_ > 5.50; see Table 1 for statistics): responses were greater, on average, with the Saccade Only condition presented first and other conditions presented in subsequent blocks. However, we found no interaction between Condition and Order in any of the ROIs, so the order of the condition blocks did not affect responses differentially across conditions. Consequently, for the subsequent analyses we analyze the data from both versions of the experiment together.

**Table 1:** **Analysis of variance (ANOVA) statistics by region of interest (ROI) for the condition order analysis of Experiment 3**.

ROI	V1v	V2v	V3v	V1d	V2d	V3d	V3A	V4	LO	OFA	FFA	OPA	PPA
*F*_(3,1) condition_	**14.51**	**22.37**	**22.41**	2.28	1.65	2.24	**13.64**	**31.44**	**13.79**	**12.31**	**18.45**	**8.26**	**23.34**
p _condition_	**0.00**	**0.00**	**0.00**	0.10	0.19	0.10	**0.00**	**0.00**	**0.00**	**0.00**	**0.00**	**0.00**	**0.00**
*F*_(3,1) order_	2.81	**7.53**	**5.61**	**9.38**	**9.48**	**5.51**	2.29	**5.56**	0.70	1.20	1.08	0.36	1.10
p _order_	0.10	**0.01**	**0.02**	**0.00**	**0.00**	**0.02**	0.14	**0.02**	0.41	0.28	0.31	0.56	0.30
*F*_(3,1) interaction_	0.96	1.94	1.25	1.41	1.16	0.67	0.43	1.53	0.49	0.33	0.80	0.25	0.16
p _interaction_	0.42	0.14	0.31	0.26	0.34	0.57	0.73	0.22	0.69	0.80	0.51	0.86	0.93

*Bold values indicate significant effects (p < 0.05). Adjacent rows shows F values for one condition (above) and the associated p values (below) for each ROI. F values are for the variables condition, order, and the Interaction between them*.

#### Stimulus Location Remapping

**Figure 7:**
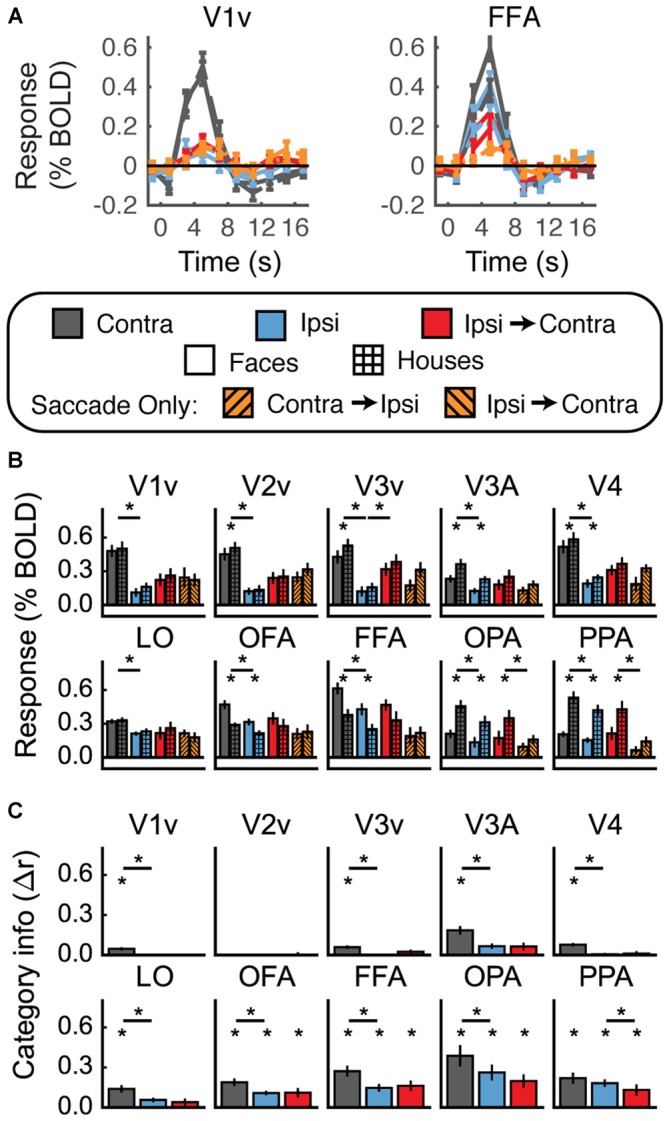
**Experiment 3 results. (A)** Estimated HRF functions for two representative ROIs (V1v and FFA). “v” Denotes the ventral portion of the ROI, which represents the upper visual field where the stimuli appeared. **(B)** Bar graphs for percent signal change per condition per ROI. The lower level of asterisks indicate significant (*p* < 0.05) differences between responses to faces and houses. Lines with asterisks above them denote significant (*p* < 0.05) differences between eye movement conditions (e.g., between Contra and Ipsi). No regions show a pattern of responses consistent with remapping of stimulus location. **(C)** Feature information (difference of *z*-scored Pearson correlations, Δr) for each ROI. Asterisks directly above the bars indicate significant feature information for that condition (Δr > 0, *p* < 0.05). Asterisks between bars indicate significant differences in feature information (*p* < 0.05). No region shows a pattern of responses consistent with remapping of feature information (an increase in feature information in the Ipsi-Contra condition vs. the Ipsi condition).

Univariate fMRI results averaged across all subjects are shown in Figures 7A,B. Unexpectedly, with the conditions blocked together and the background substantially darkened, none of the visual areas showed a pattern of responses consistent with remapping. The mean response in the Ipsi-to-Contra condition was not significantly larger than the mean response in the Ipsi condition in any region except V3v, and there the response in the Ipsi-to-Contra condition was not significantly larger than in the Saccade Only condition. The lack of a difference between the Ipsi-to-Contra and Ipsi conditions may have been due to larger responses in the Ipsi condition than were measured in the previous experiments (compare blue bars in Figure 7B to the same bars in Figures 4B, 5B).

**Figure 8:**
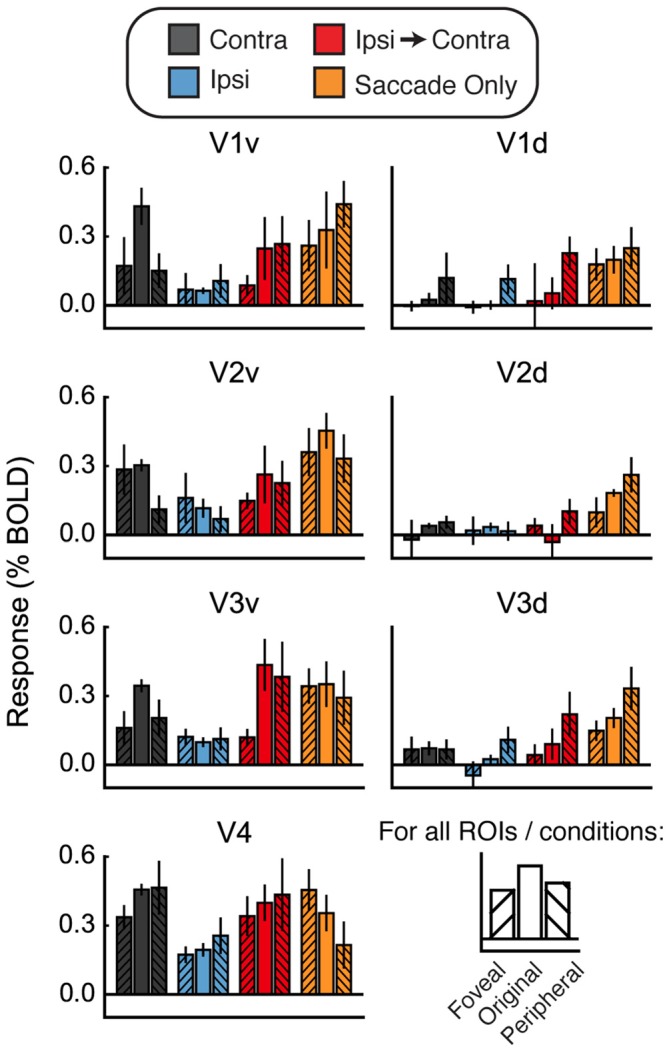
**Foveal/peripheral ROI analysis for Experiment 3.** Responses within the foveal, original, and peripheral ROIs. Responses to faces and houses are averaged in each bar. In the Contra condition, responses are largest in the original ventral ROIs, but the foveal and peripheral ROIs also respond more than in Experiment 2. Responses in the Ipsi condition are also above zero. Both the Ipsi responses and the foveal/peripheral responses are likely caused by diffuse lightening of the upper part of the screen at stimulus onset. Once again, in the Ipsi-to-Contra condition and the Saccade Only condition, responses in the peripheral ROIs are approximately as large as responses in the original ROIs. These peripheral responses suggest that the responses in the original ROIs may not be due to remapping of stimulus-specific location (or other) information.

One possibility is that the appearance of a bright stimulus image on the darkened screen may have brightened the whole scanning environment enough to elicit a small response in regions representing the entire visual field. To determine whether the increased Ipsi responses were due to diffuse activation in response to the bright flash of the stimulus, we performed the same foveal/peripheral ROI analysis as in Figure 6 with the data from Experiment 3. Results of this analysis are shown in Figure 8. As in Experiment 2, the largest responses in the Contra condition were observed in the original (central) ROIs. However, unlike Experiment 2, the responses in the foveal and peripheral ROIs were increased above baseline in V1v, V2v, and V3v. This suggests that the bright stimulus onset may have elicited a diffuse response across all the visual areas responding to the top half of the screen (the bottom half was substantially dimmed by a neutral-density filter—see “Materials and Methods” Section).

As in Experiment 2, responses in the Ipsi-to-Contra condition were not confined to the original ROIs. Responses in both the Ipsi-to-Contra and Saccade Only conditions in the peripheral ROIs were as large as responses to those conditions in the original ROIs. However, in V1 in particular, the responses in the peripheral ROIs in the Ipsi-to-Contra and Saccade Only conditions appeared diminished compared to Experiment 2. The smaller responses in the periphery were likely due to the darkened scanning environment, and a consequently smaller difference between the edge of the projector screen and the scanner bore behind it. The asymmetry between the two saccade directions was not present in as many regions in Experiment 3 as it was in Experiment 2, which is also consistent with a reduced effect of illumination differences in the periphery. Even though the responses in the Saccade Only condition appear to be less influenced by luminance-related factors in Experiment 3, the responses to the Saccade Only condition (in foveal, original, and peripheral ROIs) were still as large as the responses in the Ipsi-to-Contra condition.

One final reason that the Ipsi responses were larger in Experiment 3 could be that subjects did not have to move their eyes at all in blocks containing the Ipsi condition in this experiment. Thus, it may have been easier for them to attend to the location of the stimulus in the Ipsi condition in this experiment vs. in previous experiments. Attention is known to modulate BOLD responses when a stimulus is expected to appear (McMains et al., 2007), so uninterrupted attention to the stimulus location may have resulted in increased responses in the Ipsi condition.

In sum, despite our attempts to match previous experimental designs as closely as possible, we once again did not measure a reliable signature of stimulus location remapping.

#### Feature Information Detection and Feature Information Remapping

Multivariate results are shown in Figure 7C. In most regions we tested (all except V2v and the dorsal parts of V1–V3) we found significant levels of feature information with contralateral stimulus presentation (Figure 7C, Supplementary Table 3). However, despite the presence of feature information in nearly all of our ROIs, none of the regions we investigated showed any evidence for remapping of stimulus feature information.

## General Discussion

The current study set out to determine what information (if any) remaps across saccades. Remapping has been suggested as a potential mechanism for visual stability and/or coordinate transformation of visual input. Remapping of spatial information has been reported using neurophysiology (Duhamel et al., 1992; Walker et al., 1995; Umeno and Goldberg, 1997; Nakamura and Colby, 2002; but see also Zirnsak et al., 2014), behavior (Rolfs et al., 2011), EEG (Bellebaum et al., 2005; Bellebaum and Daum, 2006), and fMRI (Merriam et al., 2003, 2007). However, it remains unresolved whether information about stimulus features is also remapped.

We attempted to address this critical question using fMRI, measuring *location* remapping with univariate analyses, and *feature* remapping with multivariate pattern analyses of stimulus feature information. We did not find any evidence for remapping of stimulus feature information, despite robust measurements of stimulus feature information in many of our ROIs during contralateral stimulus presentation (Figures 4C, 5C, 7C). However—unexpectedly—we also did not find evidence for location remapping. Very few of the regions that we examined showed significantly larger responses to ipsilateral stimuli followed by saccades (Ipsi-to-Contra condition) than to saccades alone (Saccade Only condition; Figures 5B, 7B). Furthermore, responses in the Ipsi-to-Contra condition were not spatially localized to the cortical region representing the remapped stimulus location. Cortical regions peripheral to the region that represented the stimulus location responded as strongly as the locations that represented the stimulus.

In our third experiment, we attempted to match the conditions of our paradigm as closely as possible (given the aims of our experiment) to previous work. We darkened the screen and fMRI environment, and manipulated the order in which subjects experienced the conditions (Experiment 3 methods). None of these manipulations resulted in clear stimulus location remapping (Figure 7, Supplementary Table 3).

### Differences from Past Research

We view our experiments as a failure to generalize rather than a failure to replicate past work, since several important differences remain between our experiments and those of Merriam et al. (2003, 2007). Our stimuli were substantially larger (3.33° vs. <2°) and closer to the fovea (3.5° vs. ~8°) than the stimuli in Merriam et al. (2003, 2007). However, visual acuity decreases rapidly outside the fovea, and data from our own pilot studies and others (Schwarzlose et al., 2008; Kravitz et al., 2010) show that patterns of responses to different stimuli become harder to distinguish as the stimulus is moved toward the visual periphery. Thus it was necessary to both make the stimuli larger and move them closer to the fovea in order to increase signal to noise for measurement of feature information. We also presented our stimuli continuously rather than flashing on and off at 6–10 Hz (Merriam et al., 2003), although it is unclear whether or how stimulus flicker might influence remapped responses. The lack of flicker may have decreased the saliency of the stimulus, which may have reduced the remapped responses (Gottlieb et al., 1998; Joiner et al., 2011).

Another potentially important difference was the relative darkness during the experiment. Recent work has shown that even moderate background illumination, as well as the presence of other objects on the screen, largely abolishes remapped responses in the superior colliculus (Churan et al., 2011). The same may be true of remapping in parietal and visual cortex as well. In Experiment 3, we attempted to make the scanning environment as dark as possible. However, our stimuli were larger than stimuli in other remapping experiments, since larger stimuli substantially improve signal to measure feature information. Thus there may still have been more global illumination in our experiment, simply due to the number of pixels illuminated by the face and house images.

Finally, our analyses (and MRI scan protocols) focused on ROIs in occipital visual cortex and ventral stream object-processing areas. Based on both prior research and pilot studies, these areas were most likely to meet all of our signal quality criteria—particularly the ability to reliably measure feature information. However, it is possible that location remapping is more reliable in regions such as IPS and FEF (Duhamel et al., 1992; Umeno and Goldberg, 2001; Merriam et al., 2003).

In summary, our work suggests that the ability to measure location remapping with fMRI depends strongly on the details of the experimental paradigm. Previous work suggests that location remapping can be reliably measured with fMRI for small, highly salient stimuli presented in the far periphery in very dark environments. Our work shows that location remapping is difficult or impossible to measure with fMRI in occipital and ventral visual areas with medium-sized stimuli appearing in perifoveal space. This is an important obstacle to studying remapping of stimulus feature information with fMRI, since feature information is increasingly difficult to measure the smaller and the more peripherally stimuli are presented. It is possible that other factors may have contributed to this discrepancy, or that technical improvements in fMRI, stimulus presentation, and/or gaze-contingent eye-tracking the in scanner may resolve some of these issues in the future.

### Implications for Remapping of Stimulus Location

Our results suggest that automatic remapping of stimulus location, at least as measured by fMRI, is a more fragile phenomenon than earlier reports have suggested. Several studies from other groups have also raised questions about the effect size and behavioral importance of automatic location remapping. Churan et al. (2011) showed that remapping in the superior colliculus can be abolished by the presence of multiple objects or even screen edges. This suggests that remapping may not occur in natural visual environments, which are often visually cluttered. Zirnsak et al. (2014) have also recently shown that neural RFs in macaque FEF shift toward the target of a saccade rather than toward the neurons’ post-saccade RF locations. These results suggest that visual space is compressed around targets of eye movements rather than automatically remapped (Zirnsak and Moore, 2014). Both the Churan and Zirnsak studies focus on regions outside visual cortex, so it is possible that remapping could still occur as the original studies suggested in parietal and low-level cortical visual regions. However, behavioral results also point to compression of visual space around saccade targets (Zirnsak et al., 2011; Zirnsak and Moore, 2014), and it has been known for a long time that that trans-saccadic memory is quite poor (Irwin, 1991; Rensink, 2002). Additionally, Deubel (2004); Deubel et al. (2010) have shown that landmarks or other objects present on a screen are more important than extra-retinal signals in estimating veridical spatiotopic position of an object. Together with our results, these studies suggest that location remapping may not be automatic or robust.

### Implications for Remapping of Stimulus Features

We failed to find any evidence of feature remapping in our experiments. This null result on its own does not constitute a strong case against feature remapping, particularly given the absence of location remapping in our paradigm. However, we also note that other reported evidence for feature remapping has been weak, indirect, or potentially attributable to remapping of attentional pointers rather than features (Irwin et al., 1988; Hayhoe et al., 1991; Melcher and Morrone, 2003; Melcher, 2005; Prime et al., 2006; Melcher, 2007; Knapen et al., 2009, 2010; Cavanagh et al., 2010; Demeyer et al., 2011; Zirnsak et al., 2011; O’Herron and von der Heydt, 2013; Harrison and Bex, 2014; Subramanian and Colby, 2014; Oostwoud Wijdenes et al., 2015). Indeed, consistent with our results, a recent behavioral study also failed to find evidence of automatic remapping of feature information, suggesting instead that feature-location binding may need to be re-established following each saccade (Shafer-Skelton et al., 2015).

Of course, we cannot rule out the possibility that feature information may remap, or otherwise be transferred, under circumstances different from those tested in this study. One possibility is suggested by the results of Zirnsak et al. (2014): feature information may be transferred during a saccade, but to neurons representing locations near the saccade target instead of to neurons representing the post-saccadic location of the stimulus. Such a transfer of information would not be “remapping” in the sense implied in the original articles, since the visual field would be warped near the target of a saccade rather than faithfully remapped.

Another possibility, supported by several recent studies, is that remapping may be linked to or dependent on top-down attention. Consistent with this possibility, Mirpour and Bisley (2012) found larger remapped responses to search targets than to distractors in macaque area LIP (Mirpour and Bisley, 2012), and Yao et al. (2016) found that remapped memory traces in macaque area MT were influenced by the location of top-down attention. Neupane et al. (2016) also found that the remapped memory trace in macaque V4 only emerges after a delay, consistent with an account of remapping that posits top-down attentional mechanisms (Neupane et al., 2016; Rolfs and Szinte, 2016). Furthermore, recent work showing that border ownership of contours is remapped across saccades in V2 (O’Herron and von der Heydt, 2013) could be interpreted as evidence for remapping of visual attention pointers, since there is a known link between mechanisms of selective attention and mechanisms of border ownership (Qiu et al., 2007).

In humans, remapping also appears to be influenced by attention and task relevance. Spatial attention may remap to spatiotopic coordinates only when task-relevant (Golomb et al., 2008), and even so, can leave behind a “retinotopic attentional trace” after the eye movement (Golomb et al., 2010) that can interfere with feature perception (Golomb et al., 2014). Other behavioral studies also support the idea that attention is related to feature remapping. For example, Henderson and Hollingworth (1999) found that trans-saccadic change detection improves if the stimulus change occurs during a saccade toward the stimulus region that is changed—i.e., if the change is the target of an attention shift. Thus, stimulus feature remapping (and remapping in general) may be dependent on mechanisms of attention allocation and/or working memory.

In our experiments we intentionally sought to minimize the effects of attention to test if feature information *automatically* remaps. We gave subjects no reason to attend to the stimulus after the saccade on any trial, and found no evidence for remapping. We note that this lack of evidence for remapping in our paradigm is consistent with the hypothesis that only attentional pointers remap (Cavanagh et al., 2010; Rolfs and Szinte, 2016). However, we also note that if top-down factors are critical, it may still be possible to detect feature remapping if subjects are given a trans-saccadic task related to the experimental stimuli. Exploration of the relationship between stimulus feature remapping and attention may prove to be fruitful ground for future experiments.

### Summary and Recommendations

In summary, we found no evidence for remapping of feature information in any region we studied, but we also found no evidence for remapping of location information: saccades alone elicited responses as large as the responses to stimuli followed by saccades. Thus, we could not rule out the possibility that all responses we measured in conditions with saccades were simply due to the saccades rather than remapping. Controls for global illumination and visibility of screen edges did not resolve the issue.

Because of the lack of location remapping, our conclusions about feature remapping are somewhat limited. However, the question of what remaps is crucial for understanding visual stability, and we report our efforts here both for the theoretical contributions as well as the practical benefit to others attempting to study this question in the future. Our findings generate several recommendations for future experiments on remapping of stimulus feature information. First, care must be taken to choose stimuli, tasks, and ROIs that will meet all of the signal quality criteria described at the beginning of this article. In particular, it may be necessary to optimize stimuli to search for feature remapping in particular areas (for example, two directions of moving dot fields or two orientations of bars might provide more reliable measurements of feature information in early visual areas). While we ultimately did not succeed in measuring feature remapping in these experiments, it remains unclear whether this was due to an inherent problem with the robustness of remapping, or if technical or methodological improvements may lead to success in future endeavors.

Additionally, to rule out potential causes for large responses to saccades alone, careful attention should be paid to the subjects’ full field of view. Different regions of the screen should not differ at all in their baseline illumination. It may also be necessary to manipulate the scanning environment to achieve wide-field visual homogeneity. Since screen edges may provide landmarks or points of reference for stimulus locations, which have been shown to affect remapping (Churan et al., 2011), in an ideal experiment the edges of the projection screen should not be visible to the subject. Finally, if the scanning environment is darkened, stimuli may need to be presented at low luminance values to avoid global lightening and consequent diffuse responses to stimulus onset.

Finally, given the failure to generalize the location remapping result with our paradigm, it will be important to better establish the circumstances and parameters under which location remapping can and cannot be detected with fMRI, to evaluate if fMRI is indeed a promising approach to this question. fMRI offers many advantages that make it a potentially powerful tool for exploring representational questions such as these, but it may be the case that advances in other techniques, such as multi-unit neurophysiology and EEG, may provide better—or at least complementary—approaches to this question.

## Author Contributions

MDL and JDG conceived and designed the experiments, collected and analyzed the data, and wrote the manuscript. NK provided conceptual input and contributed to writing the manuscript.

## Funding

This work was supported by NIH grant F32EY021710 to MDL; NIH R01-EY025648, NIH F32-EY020157, and Alfred P. Sloan Foundation BR-2014-098 to JDG; NIH R01-EY13455 to NK, and the Athinoula A. Martinos Imaging Center at the McGovern Institute for Brain Research, Massachusetts Institute of Technology.

## Conflict of Interest Statement

The authors declare that the research was conducted in the absence of any commercial or financial relationships that could be construed as a potential conflict of interest.

## Footnotes

^1^http://faces.mpib-berlin.mpg.de/album/escidoc:57488
