# Supplementary material for: No Evidence for Automatic Remapping of Stimulus Features or Location Found with fMRI
Source: Front Syst Neurosci. 2016 Jun 13;10:53. doi: 10.3389/fnsys.2016.00053 (PMC4904027; doi:10.3389/fnsys.2016.00053)
Supplement: Supplementary file 1 [file DataSheet_1.pdf]

No evidence for automatic remapping of stimulus features or location found with fMRI

Lescroart, M.D., Kanwisher, N., & Golomb, J.D.

Supplementary Materials

Contents

Gabor wavelet similarity analysis

Supplementary Figure S01: Gabor wavelet similarity of faces and houses

Eye Tracking Results

Supplementary Figure S02: Eye traces for subjects from Experiments 2 and 3

Supplementary Table 1: Experiment 1 statistics

Supplementary Figure S03: Experiment 1 results with only clean eye data

Supplementary Table 2: Experiment 2 statistics

Supplementary Figure S04: Experiment 2 results with only clean eye data

Supplementary Table 3: : Experiment 3 statistics

Supplementary Figure S05: Experiment 3 results with only clean eye data

Gabor wavelet similarity analysis

To assess the how similar our face and house stimuli were in low-level features, we processed each face and house stimulus image using a set of Gabor wavelet filters. Each filter quantifies luminance variance at a particular orientation and spatial frequency. This feature space is identical to the static Gabor feature space used in Nishimoto et al (2011). Briefly, the pyramid of Gabor filters had five sizes/spatial frequencies (2, 4, 8, 16, and 32 cycles / image), four orientations (0, 45, 90, and 135 degrees), and 225 unique image locations (a 15x15 grid, for the highest spatial frequency). The filters were positioned on a square grid (with differently spaced grids for different sizes/spatial frequencies) that tiled the space of the stimulus images. There were 1,425 Gabor wavelets in total; thus each image was transformed into a vector of 1,425 values. For full details of the Gabor wavelet transform, see Nishimoto et al (2011).

To assess the low-level feature similarity of the face and house images, we computed the distance between the Gabor wavelet representations of each image. We used one minus the Pearson correlation between Gabor wavelet vectors as a distance metric. We then computed classical multi-dimensional scaling (MDS; Kruskal & Wish, 1978; Shepard, 1980) on the resulting distance matrix (criterion=metric stress). The solution for this MDS is plotted in Figure S01. The faces and houses segregate into clearly distinct groups, demonstrating that the two categories indeed do differ in low-level features.

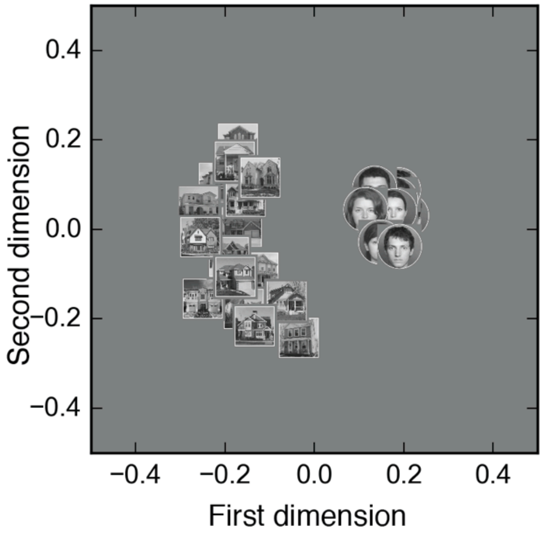

Figure S01: Gabor wavelet similarity of face and house stimuli. This is a 2D multi-dimensional scaling plot of the similarity space of our stimuli. The grouping of faces and houses into separate clusters indicates that each category is self-similar and different from the other category in the low-level features of spatial frequency and orientation.

Eye Tracking Results

Eye traces for one subject from Experiment 2 and one subject from Experiment 3 are shown in Figure S01. These subjects had reliable eye tracking, and the eye traces confirm successful execution of the fixation and saccade tasks. For some subjects, eye tracking was substantially noisier (see Methods). Overall, 54% (3627/6720), 65% (2064/3197), and 58% (6351/10,992) of the trials across all subjects in Experiments 1, 2, and 3 were retained for eye tracking analysis. For each experiment, 5/9 (Experiment 1), 3/4 (Experiment 2) and 6/11 subjects (Experiment 3—2/6 in the first half and 4/5 in the second half) had a sufficient number of retained eye trials per condition to support fMRI analysis of only trials with demonstrably good eye behavior. Excluding trials with potentially poor eye behavior gave highly similar results to those reported in the main text (compare Supplementary Figures S01, S02, and S03 with main figures 4, 5, and 7).

For trials requiring saccades, average latency (± standard deviation) from saccade cue to saccade onset in each experiment was 272 ± 104 ms, 456 ± 126 ms, and 279 ± 121 ms. The slower saccades in Experiment 2 were likely due to a change in task and in saccade cue timing from Experiment 1.

The task in Experiment 2 (detect old faces or houses) required that the subjects process the content of the stimulus rather than simply detect a salient overlaid dot. This was intended to be more demanding, and as such it likely slowed saccade response times. To compensate for the increased task difficulty, we presented the saccade cue 100 ms prior to the offset of the stimulus image instead of exactly at image offset in Experiment 2. However, three of the four subjects in Experiment 2 had been trained on the previous version of the experiment with the saccade cue exactly at image offset. These subjects were given no explicit instruction to initiate saccades immediately upon hearing the tone, so it is likely that their past experience with the task led them to wait until stimulus offset to initiate their saccades.

The subjects in the first half of Experiment 3 seem to bear out this hypothesis: the subjects with prior training on the task (n=3) were somewhat slower to initiate saccades (latency 406 ± 179 ms) than the naïve subjects (n=3; latency 332 ± 187 ms). (Saccade cue timing was the same in Experiment 3 as it was in Experiment 2.)

In all three experiments, the latency from image offset to saccade onset (272 ± 104 ms, 356 ± 126 ms, and 179 ± 121 ms) was within the window for memory trace remapping (Duhamel et al., 1992), and comparable to image offset to saccade onset latencies in previous fMRI remapping studies (Merriam et al., 2003, 2007).

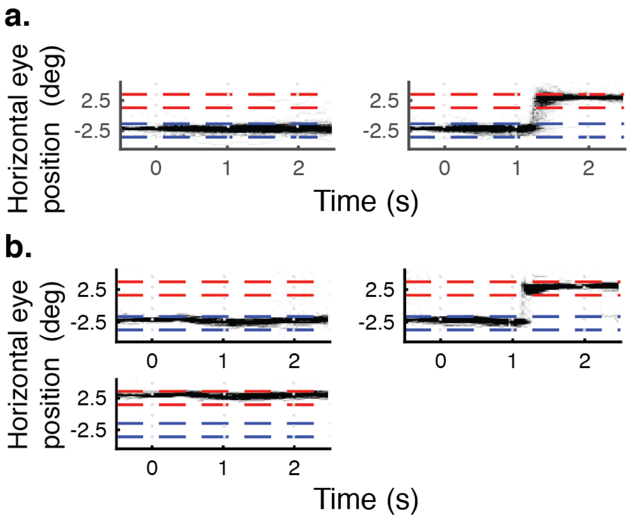

Figure S02: Eye traces for subjects in Experiments 2 and 3 (as Figure 3 in the main text). **a.** Horizontal (x) eye position for two different eye movement trial types for a single subject in Experiment 2. (In Experiment 2, each individual subject always started with eyes fixated on the same side of the screen.) To the left is fixate left and to the right is saccade left-to-right. Each plot is a density plot containing trials for both face and house stimuli. The darkness of each pixel reflects the number of trials (up to a max of 30) in which the subject's eyes were focused on a given x location during each 30 ms window. Blue and red dashed lines indicate left and right fixation targets +/-1°. Stimulus onset is at 0 s and offset is at 1 s. Eye behavior was accurate and consistent across trials. **b.** Horizontal eye position for three different eye movement trial types for a single subject in Experiment 3. Top left is fixate left, bottom left is fixate right, and top right is saccade left-to-right. Plotting conventions as in a.

Univariate Response Differences (% signal change)

ROI	V1v	V2v	V3v	V1d	V2d	V3d	V3A	V4	LO	OFA	FFA	OPA	PPA
dof	8	8	8	8	8	8	8	8	8	7	5	8	8
$t_{\text{Contra > Ipsi}}$	**8.32**	**5.69**	**8.93**	1.86	1.17	-0.35	**2.69**	**13.90**	**2.72**	**3.54**	**6.16**	**3.19**	**3.53**
$p_{\text{Contra > Ipsi}}$	**0.00**	**0.00**	**0.00**	0.10	0.28	0.73	**0.03**	**0.00**	**0.03**	**0.01**	**0.00**	**0.01**	**0.01**
$t_{\text{Ipsi-Contra > Ipsi}}$	**11.51**	**7.93**	**6.07**	**7.31**	**3.26**	2.26	**2.86**	**5.68**	**4.06**	1.92	**3.65**	1.89	1.94
$p_{\text{Ipsi-Contra>Ipsi}}$	**0.00**	**0.00**	**0.00**	**0.00**	**0.01**	0.05	**0.02**	**0.00**	**0.00**	0.10	**0.01**	0.10	0.09
$t_{\text{Face > House}}$	-1.81	**-2.63**	**-2.76**	-0.39	-0.57	0.90	**-3.34**	**-3.53**	-0.17	**2.88**	**2.94**	**-6.43**	**-8.81**
$p_{\text{Face > House}}$	0.11	**0.03**	**0.02**	0.70	0.58	0.39	**0.01**	**0.01**	0.87	**0.02**	**0.03**	**0.00**	**0.00**

Multivariate Category Info (Δr)

ROI	V1v	V2v	V3v	V1d	V2d	V3d	V3A	V4	LO	OFA	FFA	OPA	PPA
dof	8	8	8	8	8	8	8	8	8	7	5	8	8
$t_{\text{Contra > Ipsi}}$	1.62	1.69	1.62	2.02	1.32	1.18	0.82	0.88	**6.11**	**3.41**	2.39	**2.68**	**3.36**
$p_{\text{Contra > Ipsi}}$	0.14	0.13	0.14	0.08	0.22	0.27	0.44	0.41	**0.00**	**0.01**	0.06	**0.03**	**0.01**
$t_{\text{Contra > 0}}$	1.95	1.02	1.59	1.65	0.93	0.47	0.54	**2.38**	**4.99**	**4.74**	**7.23**	**4.00**	**6.03**
$p_{\text{Contra > 0}}$	0.09	0.34	0.15	0.14	0.38	0.65	0.61	**0.04**	**0.00**	**0.00**	**0.00**	**0.00**	**0.00**
$t_{\text{Ipsi > 0}}$	0.90	-0.19	-0.08	-0.67	-1.42	-0.93	-0.56	1.51	-0.09	2.28	**4.06**	**3.06**	**2.86**
$p_{\text{Ipsi>0}}$	0.40	0.86	0.94	0.52	0.19	0.38	0.59	0.17	0.93	0.06	**0.01**	**0.02**	**0.02**
$t_{\text{Ipsi-Contra > 0}}$	0.47	0.46	0.71	-0.22	-0.17	0.26	0.89	0.50	-0.12	**3.05**	**3.70**	1.41	1.96
$p_{\text{Ipsi-Contra > 0}}$	0.65	0.66	0.50	0.83	0.87	0.80	0.40	0.63	0.91	**0.02**	**0.01**	0.20	0.09
$t_{\text{Ipsi-Contra > Ipsi}}$	-0.27	0.33	0.41	0.26	0.74	0.94	1.02	-0.67	-0.03	0.06	-1.69	-1.20	-0.62
$p_{\text{Ipsi-Contra > Ipsi}}$	0.79	0.75	0.69	0.80	0.48	0.37	0.34	0.52	0.98	0.95	0.15	0.27	0.56

Remapping signal quality criterion | **Remapping Evidence**

Supplementary Table 1: Experiment statistics by ROI for Experiment 1. The upper part of the graph shows univariate statistics per ROI per contrast. The lower part of the graph shows multivariate statistics (contrasts of feature information, Δr) per ROI per contrast. The row labeled *dof* indicates the degrees of freedom for the contrasts in each column. For the whole graph, adjacent rows show t and associated p statistics for each contrast indicated (for example, $p_{\text{contra > ipsi}}$ is associated with $t_{\text{contra > Ipsi}}$ in the row above it). Bold numbers indicate a significant contrast ($p < 0.05$), gray shading indicates that the contrast in a cell fulfills a remapping signal quality criterion. Yellow shading in a cell indicates that the contrast in that cell provides evidence for remapping of feature information. (There are no yellow cells.)

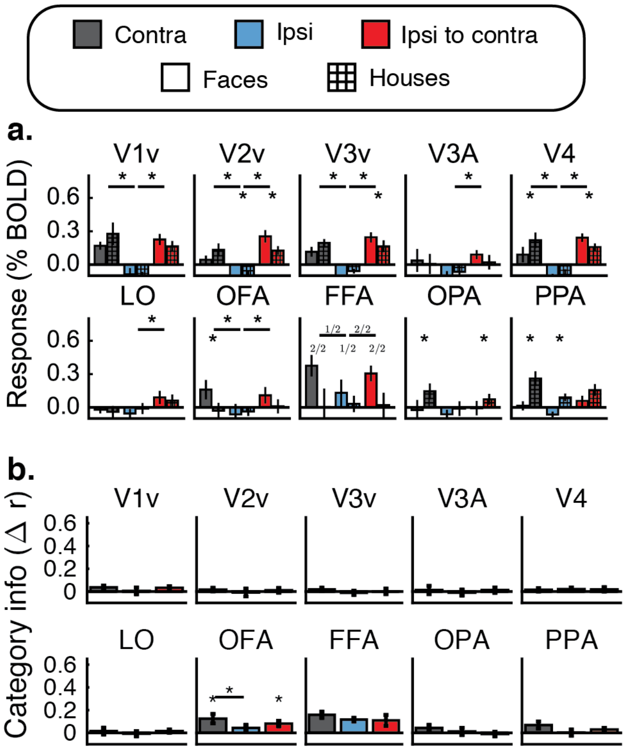

Supplementary Figure S03: Results for Experiment 1, with all trials with potentially poor eye behavior removed (compare to main text Figure 3c,d). **a.** Bar graphs for percent signal change per condition per ROI. The lower level of asterisks indicate significant (*p*<0.05) differences between face and house responses. Lines with asterisks above them denote significant (*p*<0.05) differences between eye movement conditions (e.g., between Contra and Ipsi). FFA was only defined for two of the five retained subjects; thus reliability of condition differences in FFA was determined using bootstrapped confidence intervals as in Experiment 2 (see Experiment 2 methods in main text). V1v-V4, LO, OFA, and FFA all show a pattern of responses consistent with remapping of stimulus location. These results are broadly consistent with the results in Figure 3c in the main text, though the smaller number of subjects and trials in this analysis decreases the reliability of some effects. **b.** Category information (difference of Pearson correlations, Δ*r*) for each ROI. Asterisks directly above the bars indicate significant category information (Δr>0, *p*<0.05). Asterisks between bars indicate significant differences in category information (*p*<0.05). No region shows a pattern of responses consistent with remapping of feature information (an increase in category information in the Ipsi-Contra condition vs. the Ipsi condition). Note that estimates of category information are likely to be lower simply due to the reduced number of trials used to estimate category information.

Univariate Response Differences (% signal change)

ROI	V1v	V2v	V3v	V1d	V2d	V3d	V3A	V4	LO	OFA	FFA	OPA	PPA
n	4.0	4.0	4.0	4.0	4.0	4.0	3.5	4.0	4.0	2.5	2.0	4.0	4.0
Contra > Ipsi*	**3**	**3**	**4**	0	0	0	1	**3**	**3**	0	0	**4**	2
Ipsi-Contra>Ipsi	**4**	**4**	**4**	**3**	**3**	2	**4**	**3**	**3**	**2**	0	**3**	2
Ipsi-Contra>Sacc	0	0	1	0	0	0	1	1	2	**2**	**2**	**3**	**3**
Face ≠ House	2	1	**3**	0	0	**3**	2	**4**	**3**	**1**	1	**4**	**4**
$Sac_{I-C} > Sac_{C-I}$*	**3**	2	**3**	0	1	1	1	1	0	0	0	1	2

Multivariate Category Info (Δ*r*)

ROI	V1v	V2v	V3v	V1d	V2d	V3d	V3A	V4	LO	OFA	FFA	OPA	PPA
n	4.0	4.0	4.0	4.0	4.0	4.0	3.5	4.0	4.0	2.5	2.0	4.0	4.0
Contra > Ipsi*	**3**	2	**3**	2	1	2	**3**	**4**	**4**	1	0	**3**	**3**
Contra > 0	1	2	**4**	0	2	2	**3**	**4**	**4**	**2**	1	**4**	**4**
Ipsi > 0	2	1	1	1	1	0	**3**	2	2	**3**	2	**3**	**4**
Ipsi-Contra > 0	0	0	2	2	1	1	2	0	2	1	2	**4**	**3**
Ipsi-Contra > Ipsi	1	0	0	2	2	0	1	0	1	1	0	1	0

Remapping signal quality criterion **Remapping Evidence**

Supplementary Table 2: Experiment statistics by ROI for Experiment 2. The upper part of the graph shows univariate results per ROI for different contrasts of percent signal change between conditions. The lower part of the graph shows multivariate results per ROI for different contrasts of feature information between conditions. Each cell shows the count of subjects for whom the difference in percent signal change or feature information for the indicated conditions was reliable (outside the 95% confidence interval for that subject). Bold numbers indicate cells for which a majority of subjects showed a reliable difference. The asterisk next to Contra > Ipsi indicates that this specific contrast was not carried out in the same voxels—it compares voxels in one hemisphere of the brain to voxels in the other hemisphere, which may have different signal quality, and is thus a sub-optimal comparison. We include it, with that caveat, for completeness. Gray shading indicates that the contrast in a cell fulfills a remapping signal quality criterion. Yellow shading in a cell indicates that the contrast in that cell provides evidence for remapping of feature information. (There are no yellow cells.)

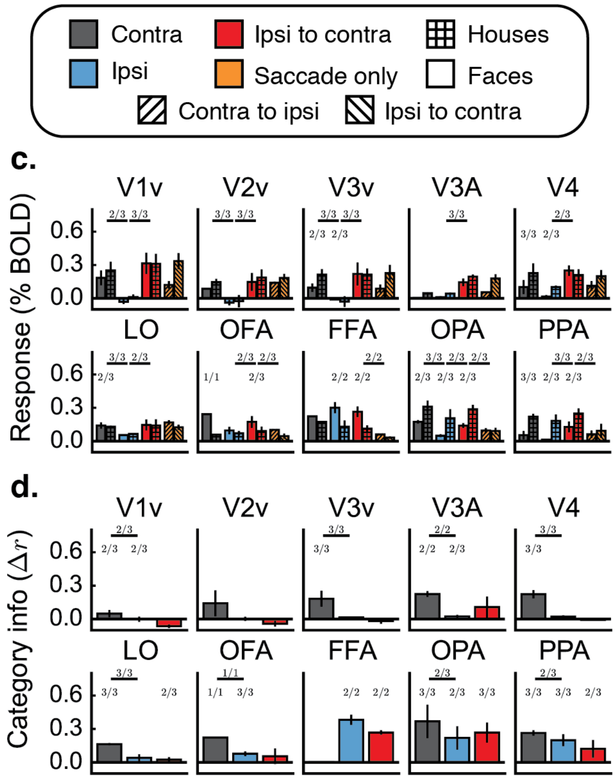

Supplementary Figure S04: Results for Experiment 2, with all trials with potentially poor eye behavior removed (compare to main text Figure 4c,d). **a.** Bar graphs for percent signal change per condition per ROI. Fractions indicate the number of subjects for whom each difference was reliable. Fractions above lines denote the number of subjects who showed reliable differences between eye movement conditions (e.g., between Contra and Ipsi). In V1v-V4, V3A, LO, OFA, and OPA, a majority of subjects show larger responses in Ipsi-to-contra condition than in the Ipsi condition. However, only in OFA, OPA, and PPA are the Ipsi-to-contra responses larger than Saccade responses in a majority of subjects. Thus, only OFA, OPA, and PPA show patterns of responses that are consistent with remapping of stimulus location. These results are broadly consistent with the results in Figure 4c in the main text, though the smaller number of subjects and trials in this analysis changes the reliability of some effects. **b.** Category information (difference of Pearson correlations, Δr) for each ROI. Fractions indicate the number of subjects for whom category information (or a difference in category information) was reliably greater than zero. No region shows a pattern of responses consistent with remapping of feature information (an increase in category information in the Ipsi-Contra condition vs. the Ipsi condition). Note that estimates of category information are likely to be lower simply due to the reduced number of trials used to estimate category information, though this problem does not affect Experiment 2 as negatively as Experiment 1, since Experiment 2 started off with many more trials per condition than Experiment 1.

Univariate Response Differences (% signal change)

ROI	V1v	V2v	V3v	V1d	V2d	V3d	V3A	V4	LO	OFA	FFA	OPA	PPA
dof	10	10	10	10	10	10	10	10	10	8	6	8	8
$t_{\text{Contra > Ipsi}}$	**8.47**	**6.22**	**6.46**	-0.03	-0.81	0.29	**6.08**	**6.34**	**6.17**	**3.31**	**3.97**	**3.11**	**4.39**
$p_{\text{Contra > Ipsi}}$	**0.00**	**0.00**	**0.00**	0.97	0.44	0.78	**0.00**	**0.00**	**0.00**	**0.01**	**0.01**	**0.01**	**0.00**
$t_{\text{Ipsi-Contra > Ipsi}}$	1.57	2.13	**3.55**	0.33	-1.09	0.24	1.10	2.21	0.31	0.89	0.36	0.85	0.61
$p_{\text{Ipsi-Contra>Ipsi}}$	0.15	0.06	**0.01**	0.75	0.30	0.82	0.30	0.05	0.76	0.40	0.73	0.42	0.56
$t_{\text{Face > House}}$	-1.00	**-2.26**	**-3.72**	0.26	0.01	-0.05	**-3.35**	**-3.14**	-0.45	**6.57**	**10.87**	**-6.35**	**-6.82**
$p_{\text{Face > House}}$	0.34	**0.05**	**0.00**	0.80	0.99	0.96	**0.01**	**0.01**	0.66	**0.00**	**0.00**	**0.00**	**0.00**
$t_{Sac\ I\text{-}C > Sac\ C\text{-}I}$	-0.42	**2.94**	**2.40**	-0.99	-1.41	0.66	1.63	**2.31**	-1.56	0.40	0.04	2.24	**2.63**
$p_{Sac\ I\text{-}C > Sac\ C\text{-}I}$	0.68	**0.01**	**0.04**	0.35	0.19	0.52	0.13	**0.05**	0.15	0.70	0.97	0.07	**0.03**

Multivariate Category Info (Δ*r*)

ROI	V1v	V2v	V3v	V1d	V2d	V3d	V3A	V4	LO	OFA	FFA	OPA	PPA
dof	10	10	10	10	10	10	10	10	10	8	5	8	7
$t_{\text{Contra > Ipsi}}$	**2.50**	0.17	**3.80**	2.09	-1.27	0.31	**4.03**	**4.87**	**3.58**	**2.52**	**5.08**	**2.34**	1.12
$p_{\text{Contra > Ipsi}}$	**0.03**	0.87	**0.00**	0.06	0.23	0.76	**0.00**	**0.00**	**0.01**	**0.04**	**0.00**	**0.05**	0.30
$t_{\text{Contra > 0}}$	**2.78**	-0.31	**2.97**	0.61	-0.57	-0.14	**3.93**	**3.87**	**3.43**	**4.46**	**4.67**	**3.50**	**3.66**
$p_{Contra > 0}$	**0.02**	0.76	**0.01**	0.55	0.58	0.89	**0.00**	**0.00**	**0.01**	**0.00**	**0.00**	**0.01**	**0.01**
$t_{\text{Ipsi > 0}}$	0.03	-0.82	-0.86	-1.65	0.24	-0.31	2.20	0.30	2.22	4.26	3.59	3.22	4.39
$p_{\text{Ipsi>0}}$	0.97	0.43	0.41	0.13	0.82	0.76	0.05	0.77	0.05	**0.00**	**0.01**	**0.01**	**0.00**
$t_{\text{Ipsi-Contra > 0}}$	**-2.37**	-0.13	1.39	0.07	0.44	0.91	2.04	0.51	1.37	**2.86**	**3.69**	**3.49**	**2.78**
$p_{\text{Ipsi-Contra > 0}}$	**0.04**	0.90	0.19	0.95	0.67	0.39	0.07	0.62	0.20	**0.02**	**0.01**	**0.01**	**0.03**
$t_{\text{Ipsi-Contra > Ipsi}}$	-1.63	0.28	2.20	1.46	0.05	1.20	-0.08	0.23	-0.66	0.11	0.12	-1.48	-5.40
$p_{\text{Ipsi-Contra > Ipsi}}$	0.13	0.79	0.05	0.18	0.96	0.26	0.94	0.82	0.52	0.91	0.91	0.18	0.00

Remapping signal quality criterion | **Remapping Evidence**

Supplementary Table 3: Experiment statistics by ROI for Experiment 3. The upper part of the graph shows univariate statistics per ROI per contrast. The lower part of the graph shows multivariate statistics per ROI per contrast. The row labeled *dof* indicates the degrees of freedom for the contrasts in each column. For the whole graph, adjacent rows show *t* and associated *p* statistics for each contrast indicated (for example, $p_{\text{Contra > Ipsi}}$ is associated with $t_{\text{Contra > Ipsi}}$ in the row above it). Bold numbers indicate a significant contrast ($p < 0.05$), gray shading indicates that the contrast in a cell fulfills a remapping signal quality criterion. Yellow shading in a cell indicates that the contrast in that cell provides evidence for remapping of feature information. (There are no yellow cells.)

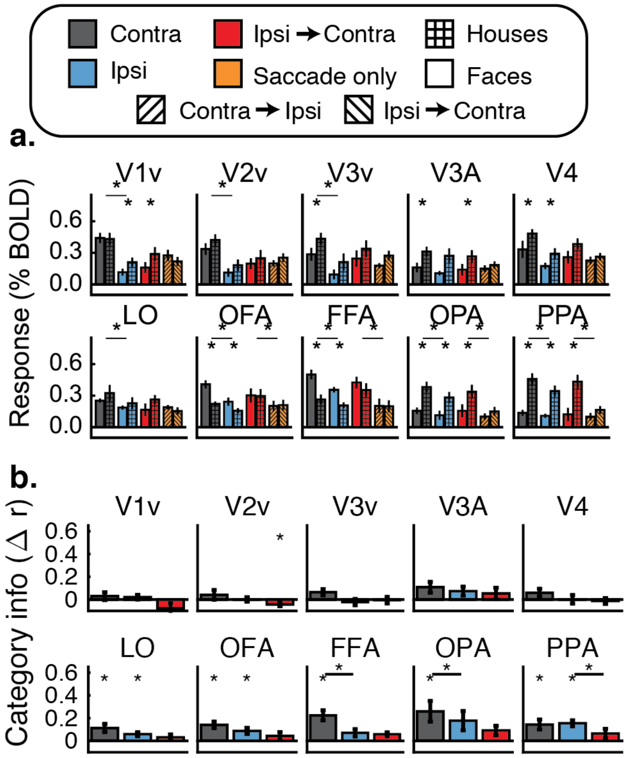

Supplementary Figure S05: Results for Experiment 3, with all trials with potentially poor eye behavior removed (compare to main text Figure 6c,d). **a.** Bar graphs for percent signal change per condition per ROI. The lower level of asterisks indicate significant ($p<0.05$) differences between face and house responses. Lines with asterisks above them denote significant ($p<0.05$) differences between eye movement conditions (e.g., between Contra and Ipsi). None of the ROIs we investigated showed a pattern indicating remapping of stimulus location. These results are broadly consistent with the results in Figure 6c in the main text, though the smaller number of subjects and trials in this analysis changes the reliability of some effects. **b.** Category information (difference of Pearson correlations, Δr) for each ROI. Asterisks directly above the bars indicate significant category information ($\Delta r>0$, $p<0.05$). Asterisks between bars indicate significant differences in category information ($p<0.05$). No region shows a pattern of responses consistent with remapping of feature information (an increase in category information in the Ipsi-Contra condition vs. the Ipsi condition). Note that estimates of category information are likely to be lower simply due to the reduced number of trials used to estimate category information.
